# QTL mapping in white spruce: gene maps and genomic regions underlying adaptive traits across pedigrees, years and environments

**DOI:** 10.1186/1471-2164-12-145

**Published:** 2011-03-10

**Authors:** Betty Pelgas, Jean Bousquet, Patrick G Meirmans, Kermit Ritland, Nathalie Isabel

**Affiliations:** 1 Natural Resources Canada, Canadian Forest Service, Laurentian Forestry Centre, Québec, Québec, G1V 4C7, Canada; 2 Arborea and Canada Research Chair in Forest and Environmental Genomics, Forest Research Centre and Institute for Systems and Integrative Biology, Université Laval, Québec, Québec, G1V OA6, Canada; 3 Current address: Institute of Biodiversity and Ecosystem Dynamics, Universiteit van Amsterdam, PO Box 94248, 1090GE Amsterdam, The Netherlands; 4 Department of Forest Science, Faculty of Forestry, The University of British Columbia, 2424 Main Mall, Vancouver, BC, V6T 1Z4, Canada

## Abstract

**Background:**

The genomic architecture of bud phenology and height growth remains poorly known in most forest trees. In non model species, QTL studies have shown limited application because most often QTL data could not be validated from one experiment to another. The aim of our study was to overcome this limitation by basing QTL detection on the construction of genetic maps highly-enriched in gene markers, and by assessing QTLs across pedigrees, years, and environments.

**Results:**

Four saturated individual linkage maps representing two unrelated mapping populations of 260 and 500 clonally replicated progeny were assembled from 471 to 570 markers, including from 283 to 451 gene SNPs obtained using a multiplexed genotyping assay. Thence, a composite linkage map was assembled with 836 gene markers.

For individual linkage maps, a total of 33 distinct quantitative trait loci (QTLs) were observed for bud flush, 52 for bud set, and 52 for height growth. For the composite map, the corresponding numbers of QTL clusters were 11, 13, and 10. About 20% of QTLs were replicated between the two mapping populations and nearly 50% revealed spatial and/or temporal stability. Three to four occurrences of overlapping QTLs between characters were noted, indicating regions with potential pleiotropic effects. Moreover, some of the genes involved in the QTLs were also underlined by recent genome scans or expression profile studies.

Overall, the proportion of phenotypic variance explained by each QTL ranged from 3.0 to 16.4% for bud flush, from 2.7 to 22.2% for bud set, and from 2.5 to 10.5% for height growth. Up to 70% of the total character variance could be accounted for by QTLs for bud flush or bud set, and up to 59% for height growth.

**Conclusions:**

This study provides a basic understanding of the genomic architecture related to bud flush, bud set, and height growth in a conifer species, and a useful indicator to compare with Angiosperms. It will serve as a basic reference to functional and association genetic studies of adaptation and growth in *Picea *taxa. The putative QTNs identified will be tested for associations in natural populations, with potential applications in molecular breeding and gene conservation programs. QTLs mapping consistently across years and environments could also be the most important targets for breeding, because they represent genomic regions that may be least affected by G × E interactions.

## Background

For genera where a reference genome is already available [1] or for others with large and unsequenced genomes such as for the vast majority of forest tree species, quantitative trait locus (QTL) mapping still represent an attractive approach that can help improve our comprehension of the genomic architecture of adaptive traits [2-4]. Under certain conditions, QTL studies also permit the identification of candidate regions for further in-depth genomic characterization [5]. In non-model plant species, there exists a vast body of literature on the genomic architecture of quantitative traits [6-9]. However, the conclusions of those studies on the number and location of QTLs are often confined to a single given experiment or experimental cross [10]. The comparison of QTLs among studies and species is also made difficult due to the large varieties of experimental procedures used to score phenotypic traits. An additional reason is that syntenic QTLs are difficult to identify and validate due to the limited number of orthologous markers commonly positioned among unrelated families or species.

With the advent of high-throughput genotyping technologies and gene catalogs, it is now possible to construct dense linkage maps with markers derived from expressed gene sequences. This new generation of gene markers transferable across pedigrees, including ESTPs, COSs and SNPs [11-14], allows the construction of consensus linkage maps with hundreds of expressed gene loci [13]. Furthermore, such gene-based markers are most often orthologous and should enable comparative mapping studies (synteny and colinearity) and subsequently, comparative analysis of QTLs among species and genera [15,16], a task that has been hardly initiated in conifers [17,18].

In conifers such as loblolly pine (*Pinus taeda *L.), maritime pine (*Pinus pinaster *Ait.), Douglas fir (*Pseudotsuga menziesii *[Mirb.] Franco) and sugi (*Cryptomeria japonica *D. Don), the main focus of QTL studies has been on economical and adaptive traits such as wood properties [8,19-24], and growth characteristics [9,25]. Up to now, few QTL studies have focused on adaptive characters tied to phenological traits and cold hardiness [26-29]. The context has recently changed, because adaptive traits are directly involved with response to climate change. Renewed interest in phenology is how it impacts on growth-related traits in plants in the context of global warming, and how it might affect plant productivity and ecosystem services [30,31].

Phenophases correspond to adaptive traits, such as bud flush and bud set, are influenced in part by heredity and by the environment [32,33]. They determine to a certain extent the fitness of individuals under particular climatic conditions [34]. Temperature is a main driver of developmental processes in plants both alone and through interactions with other environmental factors, such as photoperiod [35,36]. Considering that many factors, such as the environment and the genetic background, affect QTL detection, QTL comparative mapping between unrelated families in different environments through time represents an interesting strategy to highlight consensus regions of importance for a given trait [18,37].

Bud phenology is known to be variable and quite highly heritable in forest trees [32,38,39]. Heritability estimates for bud flush may range from 0.44 to 0.98 [33,40-44] while for bud set, heritability values may range from 0.37 to 0.72 [33,40]. For height growth, heritability can reach up to 0.86 [45,46]. Moreover, some genetic studies on poplar (*Populus sp*) and willow (*Salix sp*) have reported that relatively few loci with major effects controlled these traits [33,41,44]. Conversely, other studies in Douglas fir and poplar have revealed that growth and bud development were subject to control by many genetic loci with small to moderate effects [1,2,26,28]. In white spruce, the number and size effects of genes underlying these adaptive traits have not been characterized yet.

Recently, genetic maps with some tens to hundreds of gene-based markers have been constructed in white spruce for different segregating families [12,13], which provides a solid foundation to investigate the genomic architecture of adaptive traits. While expanding these maps with additional gene-based SNPs, the specific objectives of the current study were: i) to identify congruent QTLs or genomic regions underlying phenological traits across different pedigrees, years, and environments and ii) to determine putative genomic regions associated in part with the photoperiodic control of phenology in white spruce. To do so, the present comparative QTL mapping study was conducted with clonally-replicated material representative of two unrelated white spruce families raised in natural outdoor and environmentally-controlled conditions, and was conducted through successive growing seasons.

## Methods

### Plant material

Two large unrelated full-sib families of white spruce (*Picea glauca *(Moench) Voss) were used separately to map QTLs. The first QTL mapping population, already used in a previous genetic mapping study [13], was cross C96-1-2856 (♀80112 × ♂80109) hereafter named cross *P*, which progeny was assessed throughout growing seasons 2004 and 2005, i.e. at the age of 2 and 3 years. The second QTL mapping population was cross C94-1-2516 (♀77111 × ♂2388) hereafter named cross *D*, which progeny were assessed throughout growing successive seasons 2005, 2006, and 2007, i.e. at the age of 3, 4 and 5 years. Progeny of both crosses were clonally propagated by root cutting and maintained at the Valcartier Experimental Station near Quebec City, Canada (VES, 46°57'2"N, 71°29'50"W; Canadian Forest Service, Natural Resources Canada) under natural outdoor conditions. During the growing seasons, trees were fertilized daily with a solution 1 g L^-1 ^of a mix 3:1 (w:w) of 20-08-20: 20-20-20 commercial fertilizers (outdoor conditions: Solutech, Coopérative Fédérée, Québec, Canada; controlled conditions: Plant-Prod, Brampton, Ontario, Canada). To promote bud set and growth cessation, fertilization was stopped during the first three weeks of August, but trees were kept well watered. Fertilization was resumed with a solution 1 g L^-1 ^of fertilizer 08-20-30 until the end of the experiment.

### Genetic maps

#### a. Development of gene based-markers and genotyping

SNP markers derived from a collection of 16,500 white spruce EST clusters previously assembled by Pavy et al. [47] were assayed on both crosses. For cross *P*, the genotyping data from a previously developed GoldenGate bead array (Illumina Inc., San Diego, California, USA) [48] of 768 SNPs representing 425 genes (Table 1, Arborea PgLM0 array) was used [13]. As well, a second set of 384 SNPs representing as many genes were genotyped for cross *P *using the GoldenGate assay developed for white spruce and also for the closely related Sitka spruce (Treenomix project, [49]), and resulted in 120 scorable and segregating SNPs in this white spruce cross. For cross *D*, a new and larger Illumina bead array of 1,536 SNPs representing 822 genes (Table 1, Arborea PgLM1 array) was specifically constructed using the GoldenGate assay. The array contained 90 SNPs derived from *in silico *identification in white spruce clusters of expressed sequence tags (ESTs) following the methods of Pavy et al. [50], and 1,416 SNPs and 30 indels (varying from 1 to 30 nucleotides) selected after genomic re-sequencing of the mapping parents and of one haploid megagemetophyte (for paralogs check) following the methods of Pavy et al. [13]. The 30 indels were located in untranslated regions. TreeSNPs was used for data management [51]. To be valid and successfully genotyped, the SNPs (including the indels) had to get a minimum quality GenTrain score of 0.25 [13] and had to segregate in the mapping population. Gene nomenclature follows the Arborea GCAT3.3 white spruce gene catalogue encompassing around 28,000 genes from the clustering of 272,172 ESTs (http://www.arborea.ulaval.ca/research/sequencing/gene_catalogue/index.html). Additional file 1 provides a list of primer sequences used for PCR amplification and unigene identifiers (https://www.gydle.com/arborea/gcat/) corresponding to the unigene sequences and their annotations from the gene assembly GCAT3.3 (http://www.arborea.ulaval.ca).

**Table 1:** Genotyping success rates obtained with the 768 and 1,536 SNPs Arborea bead arrays for each of two white spruce QTL mapping populations using the GoldenGate assay.

Bead array (cross)	Number of SNPs (percent success rate)			Number of genes (percent success rate)	
	
	SNPs assayed	SNPs with GenTrain score **≥ 0.25 **^**a**^	Segregating SNPs with GenTrain **score ≥ 0.25 **^**a**^	Genes assayed	Genes with segregating SNPs
Arborea PgLM0 (for mapping cross *P*) ^b^					
Total	768	603 (79%)	516 (67%)	425	330 (78%)
Number of resequenced SNPs ^c^	730	572 (78%)	505 (69%)		
Number of *in silico *SNPs ^d^	38	31 (82%)	11 (30%)		
Arborea PgLM1 (for mapping cross *D*)					
Total	1,536	1,261 (82%)	1,100 (72%)	822 ^e^	672 ^f ^(82%)
Number of resequenced SNPs ^b ^	1,416	1,159 (82%)	1,051 (74%)		
Number of resequenced indels ^g^	30	21 (70%)	21 (70%)		
Number of *in silico *SNPs ^c^	90	77 (86%)	28 (31%)		

^a ^For SNPs with a GenTrain score ≥ 0.25, valid GenCall scores were obtained for 99.5% of samples, on average.

^b ^As reported by Pavy et al. [13].

^c ^Percentage obtained by using the total of 768 SNPs assayed per cross or the total number of genes assayed as a reference.

^d ^*in silico *SNPs were detected in clusters of aligned EST sequences derived from white spruce cDNA libraries from various individuals but not implicating the mapping parents Pavy et al. [50], which explains the lower rates of segregating SNPs obtained.

^e ^including 330 genes shared with PgLM0.

^f ^including 250 genes shared with PgLM0.

^g ^Including 21 gene indels of 1 to 2 bp, 6 indels of 3 to 6 bp, and 3 indels of over 10 bp, from untranslated regions.

#### b. Map assembly

For each QTL mapping population *P *and *D*, two individual (parental) and one sub-composite linkage maps were assembled from 260 and 500 progeny, respectively, using the ''two-way pseudo-testcross'' strategy [52]. The construction of these maps and the assembly of a composite map representative of the white spruce genome were carried out according to the procedures outlined by Pelgas et al. [11]. Marker grouping and linked loci ordering were performed using a minimum LOD threshold value of 6.0 and a minimum recombination fraction (θ) of 0.35.

### Experimental design and treatments

Six ramets for each of 395 and 740 progeny from the QTL mapping populations *P *and *D*, respectively, with one ramet placed randomly within each of six blocks (three "indoor" and three "outdoor" blocks), were maintained over several growing seasons. Growth cessation and bud set occurred under either natural outdoor conditions at VES for the three outdoor blocks, or under environmentally-controlled conditions at Agriculture and Agri-Food Canada (AAFC, in Quebec City, Canada) for the three indoor blocks. At summer solstice (21^st ^June), indoor blocks were transferred at AAFC in a large growth chamber and exposed to a declining photoperiod to simulate natural changes occurring at VES in order to help identify QTL clusters responding specifically to photoperiod. For that purpose, the photoperiod inside the growth room was adjusted weekly from the summer solstice to the end of September, i.e. from 16.0 to 12.2 hours. The environmental conditions in the large growth chamber were: day/night-time temperatures of 24/15C; photosynthetic photon flux density of 600-800 mol photons m^-2 ^s^-1 ^provided by a mixture of high pressure sodium and metal halide 400W lamps (PL light Systems, Beamsville, Ontario, Canada). Measures of bud flush were completed at VES in the spring for all blocks just before the moving of three of them to AAFC (indoor conditions). Inversely, measures of bud set were completed for three blocks at AAFC just before the moving to VES in the fall. For both QTL mapping populations (crosses *P *and *D*), outdoor and indoor blocks were considered separately during data collection and for subsequent statistical analyses.

### Phenotypic data collection

#### a. Timing of bud flush

The timing of bud flush of the terminal bud of the shoot leader was assessed three times per week by visual inspection, during two successive springs for both QTL mapping populations: in 2004 and 2005 for population *P *and in 2006 and 2007 for population *D*. Seven stages scoring the bud flushing from 0 to 6 were delineated: stage 0 being the terminal bud completely closed and stage 6 being the bud completely flushed with needles elongating [53]. At each date of bud visual inspection, bud development stage (from 0 to 6) was recorded for each tree under outdoor conditions at VES for both outdoor and indoor blocks.

#### b. Timing of bud set

The timing of bud set of the terminal bud was assessed once a week during both summers 2004 and 2005 for QTL mapping population *P*, and once per week during the summer 2006 for population *D*. For the latter population, the data were also collected two to three times per week during the 2007 growing season. Six stages of bud set were defined from 0 to 5 [53]: stage 0 being the very initiation with small white-green bud and stage 5 being the brown bud completely set with the needle fan opened towards the outside [53,54]. Data for bud set were collected at VES for outdoor blocks and at AAFC under controlled conditions for indoor blocks. At each date of bud visual inspection, the stage associated to bud formation (from 0 to 5) was recorded for each tree positioned under outdoor or indoor conditions.

#### c. Growth

For both QTL mapping populations, annual growth expressed as the total height (mm) of the current year stem was measured at the end of each growing season, once the terminal bud was set: in 2004 and 2005 for population *P *and in 2005, 2006, and 2007 for population *D*. For this latter population, the total height of each tree was also measured in 2006.

### Statistical analyses

For each phenotypic trait, statistical analyses were performed similarly for both mapping populations. Descriptive statistics and correlations were calculated from raw data for growth traits, whereas for the timings of bud flush and bud set, phenological data scores gathered for each tree were considered as the date (in Julian days) to reach each bud development stage. Then, to consider at once the various qualitative scores of bud flush or bud set datasets, principal component analysis was conducted for each of these characters at each site and each year before undertaking QTL analyses (see below).

#### a. Quantitative data analyses and correlations among phenotypic traits

Descriptive statistical parameters (Additional file 2), variances and normality (Kolmogorov-Smirnov test) of phenotypic traits were obtained using UNIVARIATE and GLM with the RANDOM option procedure of SAS (Statistical Analysis System, version 9.1.3, SAS Institute Inc., Cary, North Carolina, USA). The PLOT (QQPlot) and NORMAL options of the UNIVARIATE procedure (SAS) were further used to judge the normality of errors. Phenotypic correlation coefficients were calculated with the CORR procedure (SAS) for all pairwise trait combinations, using either Pearson or Spearman correlations, when data followed or not a normal distribution, respectively. Correlations for bud flush and bud set were estimated based on each developmental stage separately and thus processed based on the number of Julian days to reach each stage.

#### b. Principal component analyses

Principal component analyses (PCA) of phenological data scores were performed separately for bud flush or bud set using the covariance matrix as a tool of linear compression on each data set (R software [55]; R Development Core Team 2008). This transformation was done on block means to decrease the number multi-data qualitative variables (phenology multi-stages) to a single, two or three quantitative variables (composite phenotypes), which may reveal different dimensions in each dataset. This procedure was preferred to the classical approach of estimating an index from the various phenological scores because it does not suppress temporal trends in each dataset. The principal components were obtained by calculating the eigenvalues of the covariance matrix, which represent the amount of variance contributed by each factor. The Kaiser criterion was applied [56] in order to retain only factors with eigenvalues greater than 1: one to three factors per trait containing the overwhelming majority of the total variance (Table 2) were retained for linkage analyses. A rotation of components was applied when required to improve component orthogonality in comparison to initial data. Rotation was done before retaining the most important components. Factorial scores derived from these components showing no departure from normality were used for QTL analyses.

**Table 2:** Proportion of the total phenotypic variance explained by each principal component (PC1, PC2 and PC3) retained for the timings of bud flush and bud set.

**Traits and sites**^**a**^	Mapping **population**^**b**^	Year	**Proportion of total variance (range of phenology stages**^**c**^**)**					
	
			PC1		PC2		PC3	
Bud flush								
VES	*P*	2004	65.4	(4.4-4.6)	9.9	(3.6-4.0)	-	
		2005	50.1	(3.9-4.9)	12.9	(2.0-2.7)	-	
	*D*	2006	62.3	(0.4-2.3)	13.6	(3.4-5.2)	-	
		2007	56.9	(1.4-3.5)	12.8	(4.2-5.7)	-	
AAFC	*P*	2004	-	-	-	-	-	
		2005	-	-	-	-	-	
	*D*	2006	66.8	(1.6-3.4)	12.5	(4.3-5.8)	-	
		2007	73.5	(3.8-5.0)	10.9	(1.1-2.5)	-	
Bud set								
VES	*P*	2004	73.4	(3.9-4.7)	14.3	(3.3-3.9)	-	
		2005	64.4	(4.3-4.5)	13.7	(1.7)	-	
	*D*	2006	73.9	(3.1-4.3)	-		-	
		2007	66.7	(3.7-4.7)	11.9	(1.9-3.0)	6.4	(1.9-2.5)
AAFC	*P*	2005	45.8	(4.0-4.7)	34.1	(2.1)	-	
	*D*	2006	80.9	(3.4-4.7)	-		-	
		2007	64.0	(0.7-3.6)	-		-	

^a ^AAFC, indoor controlled conditions at Agriculture and Agri-Food Canada; VES, natural outdoor conditions at Valcartier Experimental Station.

^b ^*P*, first mapping population (cross C96-1-2856); *D*, second mapping population (cross C94-1-2516).

^c ^corresponds to the range of bud phenology stages explained by each principal component (indeed, PCA were performed from means of stages of three blocks for each environmental condition).

#### c. QTL analyses

Analyses were performed on all datasets separately to assess QTL consistency over mapping populations, years, and environmental conditions. Thus, single QTLs were identified. For height annual growth, clonal means across replications were taken into account separately for each year and each environmental condition of both mapping populations and it was the same for the total height data recorded only in 2006 for the population *D*. For bud flush and bud set, factorial scores derived from components retained after PCA were used as phenotypic data for each tree. Associations between segregating genetic markers and the phenotypic variability of each trait were determined for each year and each environmental condition for each individual parental map. An interval mapping approach [57] was used to first seek putative QTLs. Permutation tests were performed to set out chromosome and genome-wide significant thresholds [58]. Single-QTL model analyses were carried out with MapQTL software, version 5.0 [59]. Each linkage group was scanned with one to five markers as maximum number of neighbouring markers and 200 iterations. Permutation tests were carried out on each linkage group to compare hypotheses H_1 _(presence of one QTL on the linkage group) *versus *H_0 _(no QTLs on the linkage group). At least 1,000 data permutations were applied to each linkage group and also genome-wide to determine the chromosome-wise and genome-wise statistical significance thresholds. Only genomic regions exceeding chromosome-wise *P *≤ 0.05 (suggestive level) or genome-wise *P *≤ 0.05 (significance level) significance were reported as supporting the existence of a QTL. Multiple-QTL model analyses were performed with MultiQTL software, version 2.6 (http://www.multiqtl.com) to increase the accuracy of the estimated QTL position. The Kosambi mapping function with the option 'marker restoration' was used to reduce the effect of missing information. Permutations tests were carried out on each linkage group to compare hypotheses H_1 _versus H_0 _and also to compare hypotheses H_2 _(presence of two QTLs on the linkage group) *versus *H_1_. The two-linked QTL model was run to prevent the spurious detection of "ghost" QTLs that can arise when two QTLs are segregating on the same linkage group [60].

#### d. QTL location

The central position of each single QTL was determined by the position where the highest LOD was reached on the linkage map, i.e QTL-LOD peak. The confidence interval in centimorgans of each QTL corresponded to a LOD score drop of 1 and 2 on either side of the likelihood peak, i.e. at -1LOD and -2LOD below the QTL-LOD peak, providing at least 95% confidence [61]. To portray single QTL locations, results were graphically converted with the MapParse 2.0 software (http://mac.softpedia.com/get/Math-Scientific/MapParse.shtml, P. Meirmans, unpublished) and MapChart [62]. A simplified visualization of single QTLs observed for the four different parental maps was plotted onto the composite genetic map. Single QTLs were projected on the composite map, using markers of left and right flanking ends of the confidence intervals shared by individual linkage maps and the composite map, by means of a homothetic function as proposed by Chardon et al. [37]. In a few cases where anchor markers displayed a discrepancy in ordering between an individual map and the composite map, the projection was processed with the next flanking markers. For each trait, single QTLs sharing orthologous markers positioned in the same genomic region at the level of the composite map and in the -1LOD confidence interval were considered as characterizing the same QTL cluster. Also, to determine whether QTLs among different traits were significantly co-located, first, the proportion of QTLs within each QTL cluster for different traits that had overlapping confidence intervals was determined. Then, QTL confidence intervals were randomized across the linkage map 1,000 times, and the distribution of the proportion of overlapping QTLs of different traits determined. If this proportion of randomized QTLs was less than the original QTL overlap 95% of the time, the co-location was deemed significant.

## Results

### Genotyping SNP-array PgLM1

For the new data generated by the 1,536-SNP genotyping array PgLM1 for the cross *D*, 72% of the polymorphisms assayed (1,100 SNPs) had a Gentrain quality score of 0.25 or more and segregated among the progeny (Table 1). They were thus deemed successful. This success rate was comparable with previous genotyping success rate on the cross *P *using a 768-SNP genotyping array (PgLM0, [13]). A number of SNPs with acceptable Gentrain score were found to be monomorphic, presumably because of the failure of one of the two allele-specific oligos (ASO) included in the GoldenGate assay [13]. For the 1,100 polymorphisms successfully genotyped and segregating, the call rate was 99.5% on average, with the lowest call rate at 95% for any given SNP (or maximum of 5% missing data). Based on the inclusion of 14 positive controls, the repeatability of the genotyping assay was estimated at 99.47%. The overall success rate was higher for resequenced SNPs than for SNPs derived from the alignment of EST sequences (Table 1). Because a subset of genes (317 out of 822) had more than one SNP genotyped, the success rate was marginally higher on a gene basis (82%) (Table 1) and when two SNPs were successfully genotyped for a given gene, the one with the least missing data was kept for gene mapping purposes. A number of small indels located in untranslated regions could be successfully genotyped using the GoldenGate assay. Fifteen out of 21 indels of 1 to 2 bp assayed could be successfully genotyped, also 6 indels of 3 to 6 pb, but none of three indels above 10 bp (12, 21, 30 bp) could be successfully genotyped. Therefore, for small indels, the genotyping success rate with the GoldenGate assay was comparable to that of SNPs.

### Genetic maps

Four saturated linkage maps were assembled from 454 to 570 markers, including 283 to 451 gene SNPs (Table 3): the parental maps for the female 80112 and the male 80109 of cross *P *were assembled each on 12 major linkage groups (LGs) by positioning respectively 549 and 570 markers, including newly genotyped gene SNPs (using the Sitka spruce/white spruce SNP array, see Methods) and previously mapped AFLPs, SSRs, ESTPs, and gene SNPs [12,13]; parental maps for the female 77111 and the male 2388 of cross *D *were assembled for each of the 12 major LGs by positioning respectively 471 and 454 markers composed of newly genotyped gene SNPs (using the PgLM1 array, see above). The alignment between each homoeologous linkage group of the four individual linkage maps could be conducted on account of common gene markers between both mapping populations. Synteny and colinearity were well conserved between individual linkage maps within the same mapping population. Indeed, marker order was the same for 159 out of 165 (96.4%) homoeologous markers between the male (80109) and female (80112) linkage maps of cross *P*, and for 244 out of 258 (94.6%) homoeologous markers between the male (2388) and the female (77111) linkage maps of cross *D*.

**Table 3:** Parameters used to assemble the individual and composite white spruce linkage maps.

Mapping parameters	Individual	maps			Composite map
		
	Cross *P*		Cross *D*		
			
	♀80112	♂80109	♀77111	♂2388	
Available markers ^a^	600	671	521	508	1,578 ^b^
Distorted markers ^c^	10	12	15	19	33
Markers without segregation distortion	590	659	506	489	1545
Assigned markers	580	650	486	470	1,342
AFLP loci	256	289	-	-	470
SSR loci	9	10	-	-	12
Gene loci					
ESTPs	22	23	20	20	33
SNPs	293	328	466	450	827 ^d^
Total	315	351	486	470	860
Positioned marker loci (%)	549 (94.7)	570 (87.7)	471 (96.9)	454 (96.6)	1,301 (96.9)
AFLP loci	239	235	-	-	453
SSR loci	8	10	-	-	12
Gene loci					
ESTPs	19	19	20	20	33
SNPs	283 ^e^	306 ^f^	451	434	803 ^g^
Total	302	325	471	454	836
					
Major linkage groups (n > 10 markers)	12 (4 ^h^)	12 (1 ^h^)	12	12	12
Minor linkage groups (3 ≤ n ≤ 10 markers)	1	0	0	0	0
Unlinked markers	10	9	20	19	58
Map length *G*_*F*_, cM (Kosambi)	2,163.6	2,276.1	2,055.6	1,700.1	2,086.8
Average map density, cM (Kosambi)	3.9	4.0	4.4	3.7	1.6
Average size for major linkage groups, cM (Kosambi)	143.3	162.6	171.3	141.7	173.9

^a ^For individual linkage maps, only markers segregating 1:1 or 1:1:1:1 were used.

^b ^256 markers were shared between the two crosses.

^c ^Bonferroni correction: *P *≤ 0.01/number of loci.

^d ^373 gene loci from Arborea PgLM0 SNP array, 339 from Arborea PgLM1 array, and 115 from Treenomix array.

^e ^215 gene loci from Arborea PgLM0 SNP array, and 68 from Treenomix array.

^f ^239 gene loci from Arborea PgLM0 SNP array, and 67 from Treenomix array.

^g ^370 gene loci from Arborea PgLM0 SNP array, 325 from Arborea PgLM1 array, and 108 from Treenomix array.

^h ^Number of linkage groups composed of 2 subgroups having more than 10 markers.

**Figure 1:**
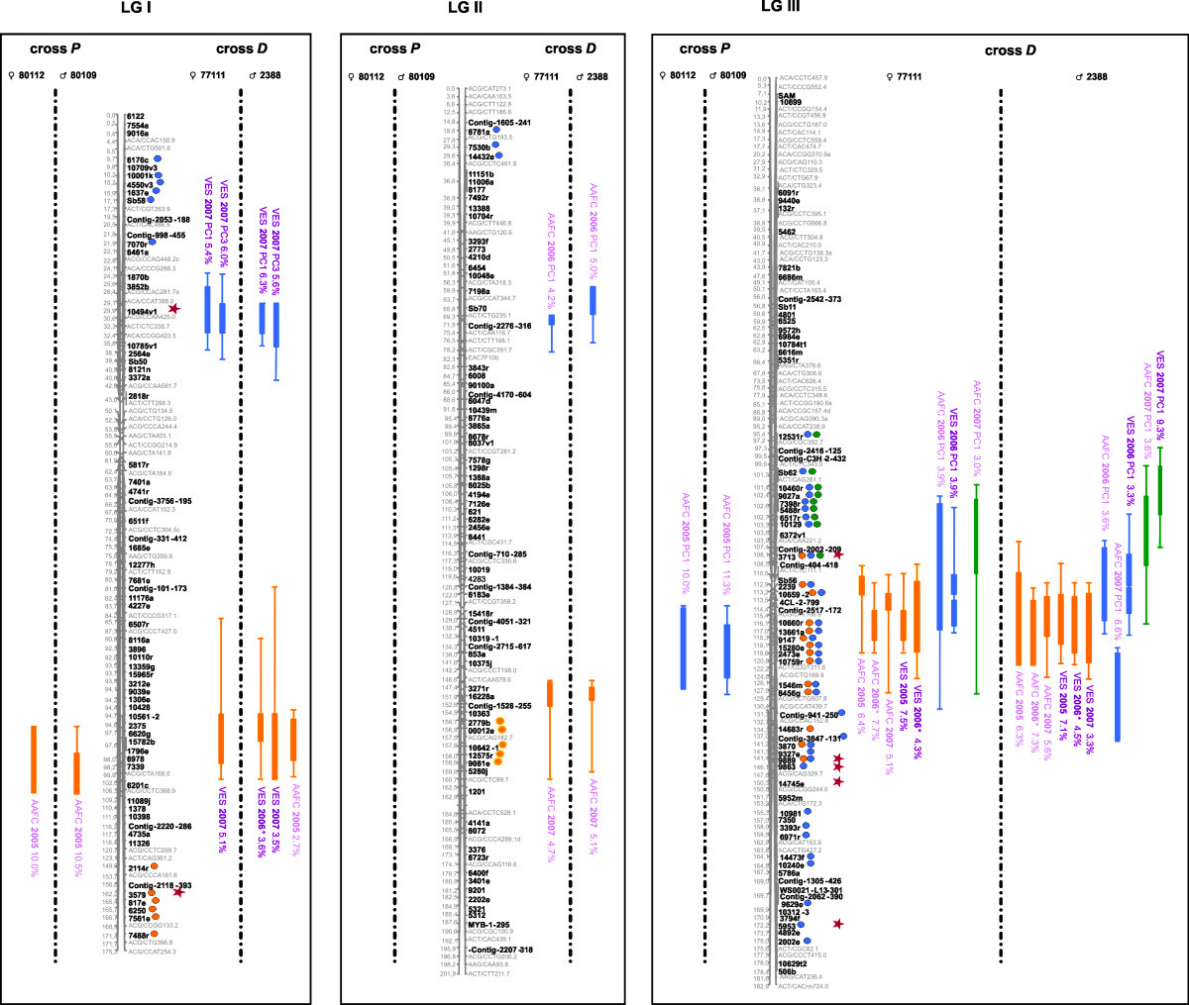
**Genomic architecture of adaptive traits in white spruce: distribution of QTLs significantly associated to bud flush, bud set, and height growth on linkage groups I to III of the composite map of white spruce assembled from both mapping populations *P *(♀ 80112 × ♂ 80109) and *D *(♀ 77111 × ♂ 2388)**. Genetic distances are indicated in cM (Kosambi) at the left of each linkage group. QTLs for bud flush, bud set and height growth are indicated by green, blue, and orange circles and bars, respectively. Thin and large vertical bars of each QTL correspond respectively to -1LOD and -2LOD confidence intervals and are positioned compared to marker position on central bar of each linkage group. Circles identify gene markers associated to each -1LOD confidence interval of each QTL. Note that because of the dense positioning of markers, physical position of circles and confidence intervals can not be plotted beside each other. QTL names correspond to environmental condition (indoor = AAFC or outdoor = VES), year and for bud flush and bud set, principal component identifying each QTL, i.e. PC1, PC2 or PC3 (Table 2). An asterisk was added for QTLs identified from total height growth in order to distinguish them to QTLs identified for annual height growth in 2006 for the cross *D*. Red stars indicate the correspondence between QTL-marker and outlier candidate gene SNPs involved in local adaption for white spruce [108].

**Figure 2:**
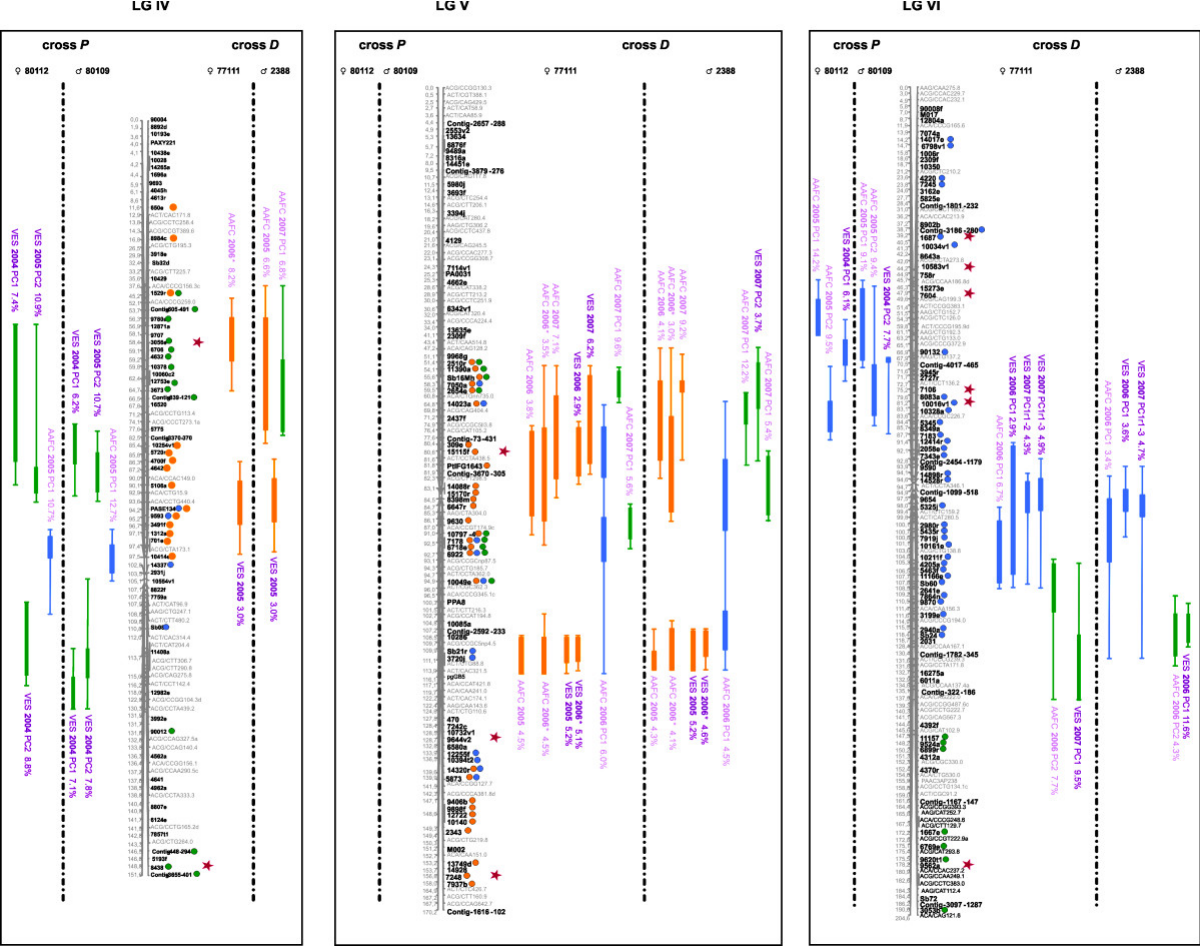
**Genomic architecture of adaptive traits in white spruce: distribution of QTLs significantly associated to bud flush, bud set, and height growth on linkage groups IV to VI of the composite map of white spruce assembled from both mapping populations *P *(♀ 80112 × ♂ 80109) and *D *(♀ 77111 × ♂ 2388)**. Genetic distances are indicated in cM (Kosambi) at the left of each linkage group. QTLs for bud flush, bud set and height growth are indicated by green, blue, and orange circles and bars, respectively. Thin and large vertical bars of each QTL correspond respectively to -1LOD and -2LOD confidence intervals and are positioned compared to marker position on central bar of each linkage group. Circles identify gene markers associated to each -1LOD confidence interval of each QTL. Note that because of the dense positioning of markers, physical position of circles and confidence intervals can not be plotted beside each other. QTL names correspond to environmental condition (indoor = AAFC or outdoor = VES), year and for bud flush and bud set, principal component identifying each QTL, i.e. PC1, PC2 or PC3 (Table 2). An asterisk was added for QTLs identified from total height growth in order to distinguish them to QTLs identified for annual height growth in 2006 for the cross *D*. Red stars indicate the correspondence between QTL-marker and outlier candidate gene SNPs involved in local adaption for white spruce [108].

**Figure 3:**
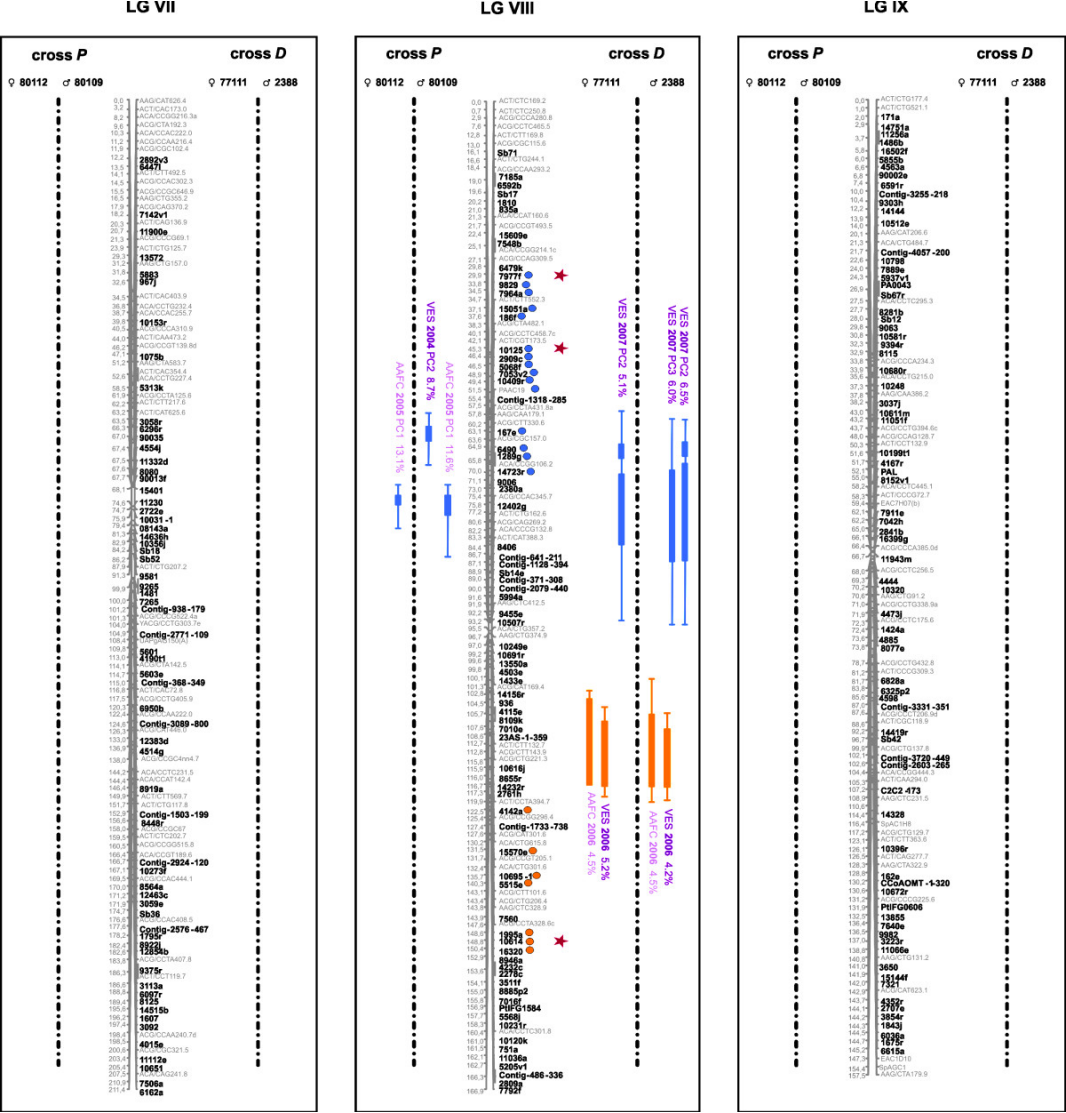
**Genomic architecture of adaptive traits in white spruce: distribution of QTLs significantly associated to bud flush, bud set, and height growth on linkage groups VII to IX of the composite map of white spruce assembled from both mapping populations *P *(♀ 80112 × ♂ 80109) and *D *(♀ 77111 × ♂ 2388)**. Genetic distances are indicated in cM (Kosambi) at the left of each linkage group. QTLs for bud flush, bud set and height growth are indicated by green, blue, and orange circles and bars, respectively. Thin and large vertical bars of each QTL correspond respectively to -1LOD and -2LOD confidence intervals and are positioned compared to marker position on central bar of each linkage group. Circles identify gene markers associated to each -1LOD confidence interval of each QTL. Note that because of the dense positioning of markers, physical position of circles and confidence intervals can not be plotted beside each other. QTL names correspond to environmental condition (indoor = AAFC or outdoor = VES), year and for bud flush and bud set, principal component identifying each QTL, i.e. PC1, PC2 or PC3 (Table 2). An asterisk was added for QTLs identified from total height growth in order to distinguish them to QTLs identified for annual height growth in 2006 for the cross *D*. Red stars indicate the correspondence between QTL-marker and outlier candidate gene SNPs involved in local adaption for white spruce [108]

**Figure 4:**
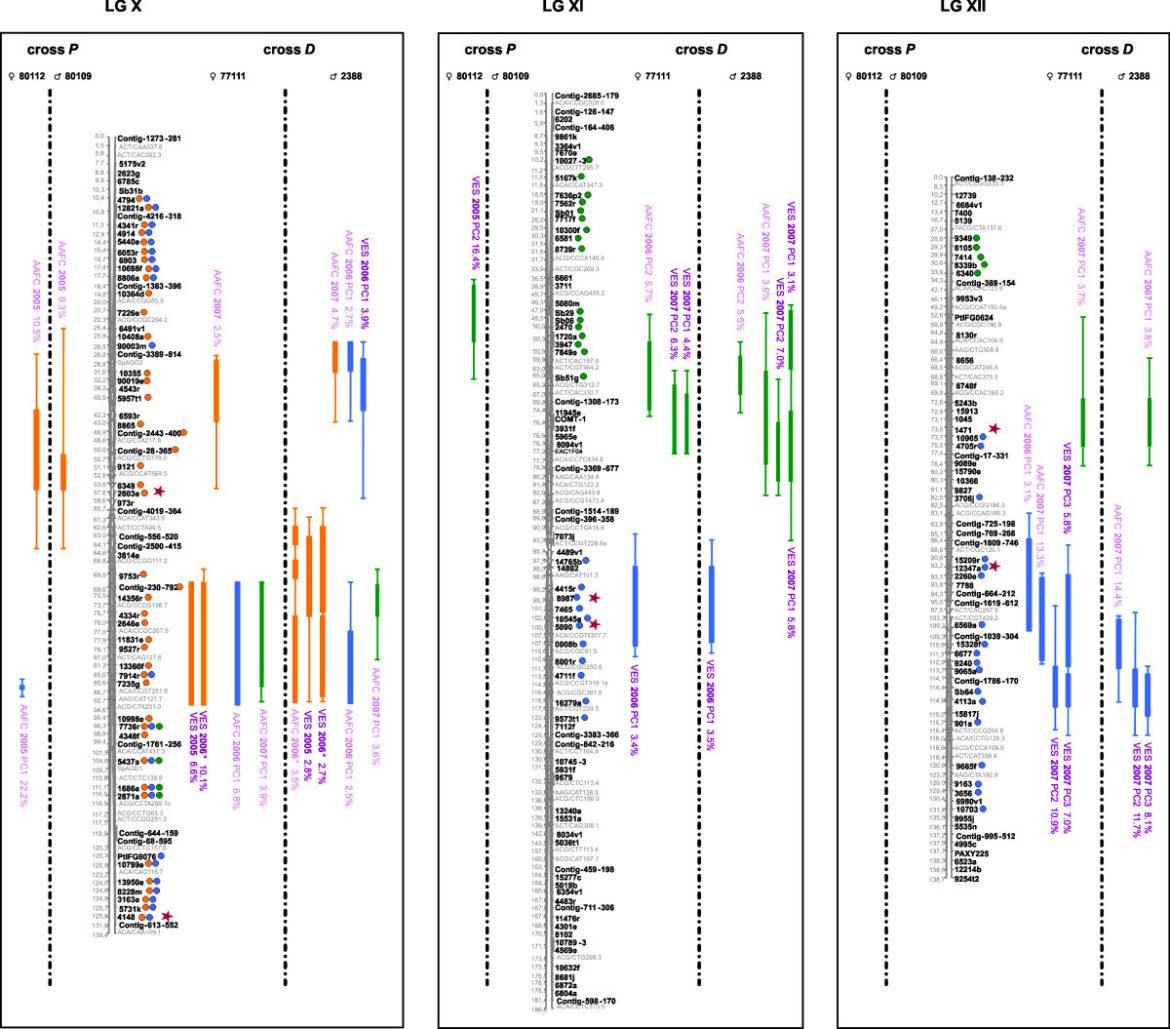
**Genomic architecture of adaptive traits in white spruce: distribution of QTLs significantly associated to bud flush, bud set, and height growth on linkage groups X to XII of the composite map of white spruce assembled from both mapping populations *P *(♀ 80112 × ♂ 80109) and *D *(♀ 77111 × ♂ 2388)**. Genetic distances are indicated in cM (Kosambi) at the left of each linkage group. QTLs for bud flush, bud set and height growth are indicated by green, blue, and orange circles and bars, respectively. Thin and large vertical bars of each QTL correspond respectively to -1LOD and -2LOD confidence intervals and are positioned compared to marker position on central bar of each linkage group. Circles identify gene markers associated to each -1LOD confidence interval of each QTL. Note that because of the dense positioning of markers, physical position of circles and confidence intervals can not be plotted beside each other. QTL names correspond to environmental condition (indoor = AAFC or outdoor = VES), year and for bud flush and bud set, principal component identifying each QTL, i.e. PC1, PC2 or PC3 (Table 2). An asterisk was added for QTLs identified from total height growth in order to distinguish them to QTLs identified for annual height growth in 2006 for the cross *D*. Red stars indicate the correspondence between QTL-marker and outlier candidate gene SNPs involved in local adaption for white spruce [108].

The sub-composite maps of crosses *P *(955 markers: 473 AFLPs, 12 SSRs, 31 ESTPs, and 439 gene SNPs including 327 SNPs from the PgLM0 array and 112 SNPs from the Sitka spruce/white spruce SNP array) and *D *(639 markers: 17 ESTPs and 622 gene SNPs from the PgLM1 array) could be merged using 251 syntenic gene loci (sub-composite maps not shown). Out of them, 221 (88.4%) were positioned in the same order through both sub-composite linkage maps, allowing to assemble the composite linkage map for the species. This composite linkage map consisted of 1,301 markers (453 AFLPs, 12 SSRs, 33 ESTPs, and 803 gene SNPs) positioned on 12 major linkage groups, corresponding to a marker density of 1 marker per 1.6 cM (Figures. 1, 2, 3 and 4, Table 3). With 836 genes, this composite map can be considered as the most enriched composite conifer map in genes up to now.

### Statistical parameters of phenotypic data

Kolmogorov-Smirnov tests and QQplots revealed that height growth data followed a normal distribution, whereas bud flush and bud set data did not always follow such a distribution except for few developmental stages. For phenotypic traits following a normal distribution, significant differences were observed among clones within cross but not among blocks. Correlation coefficients revealed a small number of significant correlations among traits ranging from low to high (Additional file 3). Pearson's and Spearman's correlation coefficients were considered as small, medium and large when values were between 0.1 to 0.3, 0.3 to 0.5, and 0.5 to 1, respectively.

### QTL detection within crosses

QTL results for each trait are tabulated in three additional files 4, 5, 6 following each individual linkage map from both mapping populations. They are also reported on Figures. 1, 2, 3 and 4 for the composite linkage map. Over all four parental maps, a total of 137 single QTLs related to growth and phenology were detected: 33 for bud flush (eight from cross *P*; 25 from cross *D*), 52 for bud set (14 from cross *P*; 38 from cross *D*) and 52 for growth (four from cross *P*; 48 from cross *D*). For bud flush, bud set, and height growth, respectively, nine, seven and 25 QTLs were significant at the 5% genome-wide level, 23, 38, and 21 were significant at the 5% chromosome-wide level (i.e. suggestive QTLs), and two, seven, and six were also considered as indicative (even if not significant). These latter trivial QTLs were retained as informative QTLs since they co-localized with at least two significant QTLs, except for one associated with only one significant QTL on LG IV for height growth (Additional file 6). For bud flush, bud set and height growth, respectively, four, six, and 11 suggestive QTLs (as defined above) co-localized with significant QTLs. Out of the total of 137 single QTLs detected, 26 (19%) were derived from the mapping population *P *and the other 111 (81%) from the mapping population *D*.

The percent of phenotypic variance accounted for by single QTLs ranged from 3.0 to 16.4% for bud flush, 2.7 to 22.2% for bud set, and 2.5 to 10.5% for height growth. The proportion of phenotypic explained variance (PPVE) for each QTL detected from cross *P *was rarely below 10% with a maximum of 22.2%, whereas for cross *D*, PPVE values were often near 5% with a maximum of 14.4%. For the first cross *P*, the sum of PPVE per individual based on the maximum LOD value was 18.5%, 45% and 19.3%, respectively for bud flush, bud set, and height growth for the parent 80109, and 36.1%, 70.2% and 21.0%, respectively, for the parent 80112, with generally two to five QTLs per trait, corresponding to an average PPVE value per QTL ranging between 9.3 and 14%. For the second cross *D*, the corresponding numbers were 69.5%, 53.6% and 51.5% for the parent 2388, and 55.0%, 55.5% and 58.6% for the parent 77111, with 9 to 11 QTLs per trait, corresponding to an average PPVE value per QTL ranging between 5.2 and 7.0%. In general for the second cross *D*, most QTLs each explained less than 10% of the total phenotypic variance on an individual basis for any of the three traits (Tables S2, S3, and S4).

### QTL comparison across individuals between crosses

At the level of the composite linkage map, a total of 34 QTL clusters (11, 13 and 10) regrouping all of 137 QTLs (33, 52, 52) were found for the three traits (bud flush, bud set, and height growth, respectively). For each trait, QTL clusters regrouped all single QTLs positioned in the same genomic regions at the level of the composite map, i.e. having shared orthologous loci in their -1LOD confidence intervals. For instance, on LG I, two QTL clusters, one for bud set and another one for height growth, were identified. The first QTL cluster regrouped four single QTLs observed with the mapping population *D*, whereas the second QTL cluster regrouped two and four single QTLs detected from both mapping populations *P *and *D*, respectively (Figures. 1, 2, 3 and 4). About one third of all QTL clusters (10 out of 34), were detected in cross *P *(20% for growth, 27% for bud flush, and 39% for bud set), compared to more than 85% in cross *D *for each trait (100% for growth, 91% for bud flush, and 85% for bud set). Indeed, the analyses performed on the mapping population *P *revealed that the 26 single QTLs mentioned above were distributed among three distinct QTL clusters for bud flush (one onto LG XI and two onto LG IV), five for bud set (LGs III, IV, VI, VIII and X), and two for height growth (LGs I and X). For each trait, bud flush, bud set, and height growth, respectively, two (67%), four (80%), and two (100%) QTL clusters were commonly identified in the two parental maps, across all years and for all tested environments. Similar analyses carried out for the mapping population *D *indicated: 10 distinct QTL clusters for bud flush (one onto LGs III, IV, X, and XII, and two onto LGs V, VI, and XI), 11 for bud set (one onto LGs I, II, III, VI, VIII, XI, and XII, and two onto LGs V and X), and 10 for height growth (one onto LGs I, II, III, and VIII, and two onto LGs IV, V, and X). For this second cross, nine (i.e. 90%), ten (i.e. 91%), and ten (i.e. 100%) QTL clusters, respectively for bud flush, bud set, and height growth, were commonly identified in the two parental maps, for all years and environments tested. By considering all 34 QTL clusters detected for the three traits on the composite map, two QTL clusters out of 11 (18%) and two out of 10 (20%) were replicated between the two mapping populations for bud flush and height growth, respectively, while three out of 13 (23%) were replicated between the two mapping populations for bud set. In fact, among the QTL clusters identified from cross *P*, 66% were also observed in cross *D *for bud flush, 60% for bud set, and 100% for height growth.

### Yearly and environmental replications of QTLs

Of the total of 10 QTL clusters identified from the mapping population *P *for the three traits, three for bud flush, five for bud set, and two for height growth, 40% (2, 2, 0) were detected in 2004 and 90% (2, 5, 2) in 2005, with 30% (1, 2, 0) shared between the two years (numbers in parentheses refer to bud flush, bud set and height growth, respectively). For this cross, comparisons between environments were limited because no measurements under controlled conditions (indoor at AAFC) could be recorded for any of the three traits during the first growing season in 2004, but bud set and height growth could be assessed under controlled indoor conditions for this mapping population in 2005. Indeed, 70% (-, 5, 2) of the 10 QTL clusters were identified during the second year (in 2005) for indoor experimentation under controlled conditions, and they were related to bud set and height growth; whereas in natural conditions (outdoor at VES), 40% (2, 2, 0) of QTL clusters were observed in 2004, and 20% (2, 0, 0) in 2005 (for bud flush, bud set, and height growth, respectively). In general, out the whole of 10 QTL clusters, 45% (-, 2, 1) were commonly detected under outdoor and indoor environmental conditions (for bud flush, bud set and height growth, respectively).

For the second mapping population (*D*), out the sum of 31 QTL clusters detected for bud flush (10), bud set (11), and height growth (10), 61% (3, 9, 7) were detected in 2006 and 61% (9, 5, 5) in 2007, with 26% (2, 3, 3) found in common between the two years. During the first growing season in 2006, 55% (3, 8, 6) of QTL clusters were detected under controlled conditions (AAFC) and 42% (7, 2, 4) in 2007 (for the three traits, respectively), whereas in natural conditions (VES), 36% of QTL clusters (1, 4, 6) were observed in 2006 and 39% (5, 4, 3) in 2007. Thus, out of 31 QTL clusters, 52% (6, 4, 6) were commonly detected from both environmental conditions for bud flush, bud set, and height growth, respectively.

When considering all mapping populations and years, QTLs were shared 64% of time (7 QTL clusters out of 11) between indoor and outdoor environments for bud flush, 46% (6 QTL clusters out of 13) for bud set, and 60% (6 QTL clusters out of 10) for height growth. Thus, more than 46% of QTL clusters were replicated into both environmental conditions. In parallel, three, five, and three QTL clusters (i.e. 32%) were identified as specific to indoor controlled conditions (AAFC) compared to one, two and one (i.e. 12%) detected only in outdoor conditions (VES), for bud flush, bud set and height growth, respectively. QTL clusters specific to indoor controlled conditions were mostly identified for the second year for bud flush (100%) and height growth (67%), whereas mainly for the first year for bud set (80%).

In general, out of the 11, 13 and 10 QTL clusters identified onto the composite map for bud flush, bud set, and height growth, respectively, 3 (27%), 6 (46%), and 6 (60%) were replicated between years. Year-specific QTL clusters were mainly observed during the first year for bud set (5 out of 7) and during the second year for bud flush (6 out of 8), and mainly for the mapping population *D*. Year-specific QTL clusters were observed as often for the first year (2 out of 10) as for the second year (2 out of 10) for height growth.

### Phenological development stages versus QTLs

For bud flush and bud set, the data used (factorial scores) for QTL analyses were generated by PCA analyses (see Methods). Considering the nature of each component retained after PCA (Table 2), each single QTL for bud set or bud flush did not necessarily correspond to only one bud development stage [53], but rather to a grouping of these different stages with variable weighting in the principal component. Thus, when considering all single QTLs characterizing each out of the 11 and 13 QTL clusters identified for bud flush and bud set, respectively, it appeared that one (onto LG VI) and two (onto LGs III and XII) QTL clusters had a group of single QTLs with components involved in almost all bud flush or bud set development stages, unlike other QTL clusters that involved principal components more narrowly defined in terms of bud development stages (Table 2). For example, the chromosomal region involved in bud set onto LG X (cross *D*: AAFC 2006 PC1, Figure 4) could play a part in the transition from stages 3 to 4 (Bud set_AAFC_cross *D*_2006_PC1, Table 2). For bud flush, the genomic region detected onto LG V (cross *D*: AAFC 2007 PC1 and VES 2007 PC2, Figure 2) could be involved into the transition from stages 4 to 5 (Bud flush_AAFC_cross *D*_2007_PC1/VES_cross *D*_2007_PC2, Table 2).

### Shared QTL clusters among traits

Permutation tests conducted onto all -1LOD and -2LOD QTL intervals (data not shown) allowed to compare QTL positions of each QTL cluster among traits and estimate the level of overlapping QTLs between phenotypic traits. Four to five occurrences of overlapping QTLs between characters considered two at a time were highly significant (with 1-P-value of 0.99 or 1.0). Five QTL clusters positioned onto LGs III, IV, V, VI, and X were identified as affecting both bud flush and bud set traits with an overlapping proportion ranging from 14.3 to 100% at -1LOD (Figures 1-2, 4). QTLs positioned onto LGs III, IV, V, and X also revealed at -1LOD a significant overlapping (from 9.1 to 100%) between QTL clusters for bud flush and height growth. QTLs detected for bud set and height growth co-located significantly onto four linkage groups (Figures 1-2, 4: LGs III, IV, V, and X). The overlapping range between QTLs affecting simultaneously height growth and bud set varied from 18.8 to 100% (Figures 1-2, 4). QTLs involving the three adaptive traits co-located together onto the linkage groups III, IV, V, and X.

To investigate associations among characters at the phenotypic level, correlations within and among the three adaptive traits were estimated and revealed some significant associations (Additional file 3). For bud flush, no or barely significant correlations for the different bud stages were observed between years for both crosses and by considering each environmental condition separately. The trend was relatively similar for bud set with a notable exception: in both crosses, a small number of low positive and significant correlations were observed under outdoor environmental conditions (VES) between stage 3 of bud set for the first experimental year and all stages of bud set for the second experimental year (from 0.192 to 0.228 for cross *P *and from 0.147 to 0.174 for cross *D*). For height growth, highly significant correlation estimates were observed between years within a range of low to medium values: under outdoor environmental conditions at VES, a moderate positive correlation was observed (0.424) between 2004 and 2005 for cross *P*, whereas for cross *D*, low positive correlations were identified (from 0.134 to 0.149) between 2005, 2006, and 2007. For this latter cross, significant correlations were observed (up to 0.340 between years) under controlled environmental conditions (at AAFC). As for relationships among traits, a small number of significant correlations ranging from low to high were identified (Additional file 3). For instance, for cross *D *in 2006, almost all stages of bud flush were slightly to highly negatively correlated with height growth (-0.179 to -0.546). In 2007, negative significant correlations were also observed between these traits (up to -0.345). These correlations reflects the observation that white spruces with an early bud flush have a longer growing season and consequently, a greater height growth. No or barely significant correlations were observed between the different stages of bud flush and bud set considering the same year of bud development. Also, almost no significant correlations were observed between height growth and bud set during the first growing season for cross *D*, whereas in 2007, low to moderate positive correlations between height growth and all stages of bud set (0.133 to 0.437) were observed. For cross *P*, correlation results were relatively similar (see details in Additional file 3), with an exception: low negative correlations were detected in 2004 between the last stages (5 and 6) of bud flush and the stages 1 and 3 of bud set (-0.184 to -0.236), suggesting that for a number of genotypes, later bud flush and earlier bud set co-occurrred, and vice-versa.

## Discussion

In white spruce, a rapidly growing number of genes (nearly 28,000) are being identified through more than 270,000 ESTs [47]. Together with SNP discovery, this resource provides the basis for highly informative QTL mapping studies associated with important adaptive traits such as bud flush, bud set and height growth, facilitating the identification of potentially causative genes in the future. In the present study, the genotyping of several hundreds gene SNPs permitted the construction of four linkage maps highly enriched in gene loci. The resulting composite linkage map of the white spruce genome represents the most dense gene-based linkage map yet for a conifer. Until recently, the restricted availability of dense marker linkage maps through several populations was a limiting factor for the accurate identification of a large number of QTLs. New high throughput genotyping methods have begun to overcome this barrier such as diversity arrays (DArT) or SNP arrays technologies, allowing the construction of high-density genetic maps with hundreds and now thousands of gene-based marker loci [13,63,64]. With the expansion of genetic linkage maps with additional gene-based SNPs, the present study highlighted the genomic architecture of the conifer white spruce for three adaptive traits by the identification of congruent QTLs underlying bud flush, bud set and height growth across different pedigrees, years, and environments.

### QTL detection

Repeatability values for bud flush (0.33 to 0.57), bud set (0.33 to 0.54), and height growth (0.49 to 0.80) indicated moderate to high heritabilities of these complex traits (detailed data not shown). These repeatability values are only suggestive, because their estimation is based on only two mapping populations and not on a large number of families. Regardless, they were about in the same order or marginally higher than those reported for similar characters in white spruce [e.g. [32]] or for other tree species [65,66]. The clonal nature of the material used herein could account for these marginally higher values. Consequently, QTLs were expected to segregate for these characters in the two mapping populations studied, as showed by others [26,28].

Of all QTL clusters observed with mapping population *P*, most (90%) were detected with population *D*. However, the opposite was not true, with only 24% of QTLs of population *D *detected in population *P*. One possible explanation for this asymmetry of QTL detection is the difference of sample size and marker density between the two mapping populations. The difference in progeny number (260 for cross *P versus *500 for cross *D*) had likely a major effect in the power to detect QTLs. Indeed, after 10 simulation tests performed with a random draw of 250 progeny at a time from 500 progeny selectioned for the cross *D*, the number of QTLs detected was in the same range than that for cross *P *(data not shown). Only 29% of all QTLs detected with cross *D *from 500 progeny were identified from 250 progeny and PPVE values were approximately twice as large as those obtained with 500 progeny. Thus, the small progeny size of cross *P *(or *D *during simulations) decreased the sensitivity of QTL detection and resulted in an overestimation of the effect size of main QTLs (PPVE >9% per QTL), while QTLs of small effects remained undetected. For Douglas fir, in the most complete QTL studies realized until now on adaptive traits in conifers across multiple years and/or multiple environments [26,28], the estimated effects of each QTL detected were relatively in the same range than that in our study using the large cross *D*, i.e. with PPVE values ranging between 1.2 to 11.5% using 98 progenies from one cross and between 0.7 to 9.5% using about 400 progenies from a second cross.

The present results corroborate previous QTL mapping simulation studies, where the use of small segregating populations (progeny size of about 200) resulted in low power detection, with disproportionately large effects on phenotype and with poor congruence of QTL position [67-70]. Although QTLs detected in the present study generally have effects in the same range as that in other forest trees [26,28,65], more QTLs of small effects (around 5%) were revealed by the larger mapping population *D *in the present study. Other QTL analyses performed with very large populations in mouse or maize also revealed that many genes of relatively small effects characterized many quantitative traits, disclosing a complex genetic architecture [70-72]. Regarding marker density, which was high in the present study, simulation studies carried out by Darvasi et al. [73] to determine the effect of marker spacing, gene effect, and population size on the power of marker/QTL association indicated that while marker density influenced very little the accuracy of QTL location, the addition of new markers supplied supplementary alleles that could be associated to the detection of new QTLs. Indeed, even with an infinite number of markers, the confidence intervals of QTLs were more strongly affected by population size and gene effect.

Another possible explanation of the lack of congruence in QTL detection between mapping populations *P *and *D *is genetic heterogeneity between populations, i.e. diverse marker subsets that would segregate in each population. Indeed, Beavis et al. [74], by observing that very few QTLs were common across four Maize populations, found that the lack of congruence among these independent linkage populations was associated to different sets of polymorphic alleles segregating in the different genetic backgrounds. In fact, polymorphic alleles at QTLs in one population can be monomorphic in another. Thus, the use of different unrelated populations increases the power to detect QTLs (see below).

### Repetition effect of experimental design

The evaluation of replicated QTLs across genetic backgrounds and environments may be essential to confirm the utility of specific marker/QTL linkage information prior to the implementation of marker-assisted breeding methods. The impact of the environment and the genetic background on quantitative trait variation and the detection of QTL has been documented early on and suggests that the general conclusions about QTLs, particularly those with small effects discovered on the basis of single environment and single population, could be erroneous [74,75]. QTLs found from experiments involving different crosses and/or different environments and/or yearly replicated can increase the number of QTLs detected and also provide additional confidence in their locations [9,26,74,76,77]. However, comparisons of QTLs can become complex due to the probability that different crosses will be segregating for different QTLs, with perhaps G x E effects, and/or a lack of common markers between crosses.

#### a. QTL stability in relation to genetic background

QTL clusters shared by the two unrelated mapping populations *P *and *D *in the present study correspond to about 20% of the total number of QTL clusters identified. This level of overlapping, in agreement with the proportion of shared gene loci between the two mapping populations, i.e. about 40%, allowed the validation of QTLs at the intraspecific level. Chromosomal regions with shared QTLs across different populations generally correspond to greater mapping precision and greater QTL significance compared to other genomic regions with QTLs discovered from single mapping populations [37,78]. In our study, syntenic regions revealed a high level of marker colinearity (> 93%) among individual linkage maps of both crosses, made easier the identification of QTLs shared between mapping populations, i.e. QTL clusters, by synteny-based projection from a homothetic function. High levels of colinearity are a prerequisite for the identification of candidate genes for traits of interest using cross-species information [79]. Meta-analyses are a good illustration of integrated QTL maps from synteny-based projections [80]. Thus, the conservation of gene order between genome maps provides a framework for the comparative analysis of genome architecture of complex traits. Cereal species, largely syntenic, lend themselves well to QTL projections across rice, maize, Sorghum, teosinte, wheat, and barley genomes [37,75,78,81,82]. For instance, rice candidate genes were projected onto the maize genome using a synteny conservation approach between maps and thence, associations between maize QTLs and genes involve in flowering time in rice were observed [37].

More comprehensive analyses of the genetic architecture of complex traits may require the consideration of multiple populations that represent a larger sample of the standing intraspecific variation and thus provide a framework for comparative analyses [80]. The existence of QTLs identified for white spruce in other *Picea *species remains to be investigated in closely-related and phylogenetically more distant conifer taxa, using homoeologous genome segments that would allow the unequivocal recognition of QTL regions across taxa. Given the high genome macro-synteny and macro-colinearity between the phylogenetically remote white spruce and black spruce [13], it is expected that a number of QTLs should be conserved across taxa. While logistically and financially demanding, such studies may also highlight the control of adaptive/wood traits by homoeologous chromosomal regions across conifer species. Recently, Casasoli et al. [16] reported a high conservation of QTLs for bud flush between two broad-leaved forest species, oak and chestnut, with the help of orthologous markers used to find homoeologous genomic regions between both species.

However, even if synteny-based projections can be useful to identify new candidate genes that explain trait variation across taxa, when orthologous QTLs underline the same trait, the involvement of each orthologous gene to the phenotype may vary across taxa. This differential contribution of one gene for a same trait between species was observed by Doust et al. [83] for the genetic control of branching in foxtail millet and in maize. As well, local gene duplications followed by subfunctionalization [84] may also contribute to differential genetic control in spite of similar QTL location. In line with this, recent comparative studies among *knox-1 *gene family members across diverse spruce species indicate that selection patterns vary among family members across different species, suggesting the differential evolution of gene duplicates among congeneric species [85]. The comparison of genes harbouring outlier SNPs putatively involved in adaptation between spruce species also suggested parallel evolution and the implication of different genes from the same gene families [86]. QTL comparative studies with more complete maps of the gene space should help understand these trends at the genome level.

#### b. QTL stability between environments

The different environmental conditions tested in the present study did not seem to influence the identification of at least half of the total number of QTL clusters. Environmental conditions differed by the fact that three blocks of progeny out of six were studied under controlled indoor conditions at AAFC (day/night temperatures fixed at 24/15°C) from the summer solstice to the end of september to evaluate the impact of the declining photoperiod on bud set compared to the three other blocks maintained under natural outdoor conditions (VES). The identification of more than 46% of QTL clusters across both environments indicates that subsets of QTLs controlling phenology and growth traits are spatially quite stable. More diverse environmental conditions remained to be tested. However, our present results are hardly comparable with those observed in Douglas fir for bud flush, which was tested under a dozen different environmental conditions, differing in elevation, latitude, moisture, temperature, day length [26,28]. QTL replications for bud flush in Douglas fir were in a range of 0% to 83% following the environmental conditions considered. However, in most cases, a very little overlap across sites was observed, suggesting that different genes controlling bud flush were expressed differently considering the diverse environments tested for Douglas fir. For bud set and growth traits, Jermstad et al. [28] observed few or no QTL replication (i.e. up to 17%) across the four different environments tested, reflecting important genotype x environment interactions. These discrepancies with the results of the present study reflect to some extent the variability of environmental conditions tested in each study but also, they correlate well with observations from analysis of quantitative genetic variation in adaptive traits in white spruce and Douglas fir. Indeed, small or null genotype x environment interactions were noted in white spruce [32], as compared to large genotype x environment interactions in Douglas fir [87,88], which could be related to more diverse environmental conditions in the Pacific Northwest, as compared to those affecting white spruce in eastern Canada [32,89-91]. In hybrid poplar (black cottonwood × eastern cottonwood), results more similar to ours with about 33% of environmental replication for bud set QTLs were observed across environments differing by temperature regimes between field and greenhouse conditions [10], suggesting that a large fraction of phenotypic variation for bud set (1/3) could be explained by a subset of stable genomic regions responding to diverse environmental conditions. Environmentally stable QTLs for bud flush and bud set in our study may correspond to genomic regions putatively involved during nearly all the stages of bud developmental processes, considering the nature of the principal components implicated in these QTLs. Thus, almost all stages of bud development may be controlled in part by genomic regions under moderate to low environmental influence. Considering the moderate to high heritability values observed for the three adaptive traits (i.e. about 0.41, 0.40 and 0.62 on average for both environments, for bud flush, bud set and height growth, respectively; detailed data not shown), these environmentally stable QTLs that explain at least 30% of the total phenotypic variance would account for more than 48% of the genetic additive variance.

#### c. QTL stability among years

Temporal stability has been observed for numerous QTL clusters. Overall, about one third of QTL clusters for bud flush were replicated across years, while it was near 50% and 60% for bud set and height growth, respectively. The repeated detection of QTLs from one year to the next suggests that several genes controlling bud flush, bud set, and height growth are repeatedly expressed through time for white spruce. QTL replication over multiple growing seasons was not always observed in previous studies [41]. However, our results are congruent with studies of bud flush in Douglas fir and for different growth traits in Maritime pine and Japanese cedar [*Cryptomeria japonica *(D. Don)] [25,26,40,92]. Another recent study revealed large temporal stability (across at least 10 years) for wood density QTLs, suggesting a link between tree maturation and temporal stability of QTLs [9]. In the present study, the information from QTLs replicated across years indicates that the phenotypic variance explained by each replicated QTL is relatively similar to that observed for year-specific QTLs for bud flush, and about 25% larger than those year-specific QTLs for bud set and height growth. These temporally stable QTLs may explain altogether at least 20%, 48%, and 32% of the total phenotypic variance for bud flush, bud set, and height growth, respectively. In view of heritability values for each trait (i.e. about 0.39, 0.40, and 0.61, on average for different years, for bud flush, bud set, and height growth, respectively; detailed data not shown), these QTLs may account for more than 51% of the genetic additive variance.

#### d. Overview of QTL stability

If our experimental design had been based on the use of only one mapping population in a single environment during one growing season, two and 13 QTL clusters would have been detected in the worse and best of cases, respectively. Such a strategy would have revealed only 6% to 38% of the total number of QTL clusters identified in the present study. By assuming a design based on only one mapping population (i.e. cross *D *because of its large progeny number) with yearly replication and testing in two the environmental conditions, up to 91% (31/34) of QTL clusters would have been identified in the present study. On the other hand, without year-to-year replication, 44% (15/34) of QTL clusters would have been observed across the two tested environments in the best case. Generally, QTL mapping designs for characters related to forest trees allowed for the detection of QTLs across different environments, but they rarely relied on replication across mapping populations and/or growing seasons [9,10,26,28,76,77]. Thus, the experimental design retained in the present study, and based on a repetition effect across mapping populations, environments, and years, was favourable to the detection of a large number of QTL clusters, in which one can observed genetic, environmental, and year-to-year stability of QTLs. In a breeding perspective, QTLs mapping consistently across years and environments could be a most important target for breeding, because they represent genomic regions that may likely be least affected by G × E interactions.

### QTL clusters associated to photoperiodic control

QTL clusters specific to natural outdoor conditions detected for bud set may highlight genomic regions that may be putatively activated by diverse connected environmental signals, mainly temperature and photoperiod. For many temperate and boreal trees including conifers such as spruces and Douglas fir, bud flush and shoot elongation are mainly triggered by rising air temperatures in early spring [93-95], whereas bud emergence and growth cessation are essentially activated by decreasing photoperiod [96,97]. In the present study, to possibly disentangle the effects of temperature and photoperiod, night and day temperatures were fixed under controlled indoor conditions, while photoperiod conditions were similar as for outdoor conditions. Moreover, soil moisture and nutrients were relatively well controlled in both environmental conditions to avoid their implication with the variation observed. Consequently, QTL clusters detected for bud set only from trees grown at AAFC indoor controlled conditions could not be influenced by temperature but mainly by photoperiod conditions. While QTL clusters specific to natural outdoor conditions for bud set may highlight only genomic regions responding simultaneously and mainly to temperature and photoperiod conditions. QTLs found specific to indoor conditions (AAFC) were mainly detected during the first year of measurement for cross *D *and the second year for cross *P*, and they were observed for composite phenotypes (after PCA) explaining the transition between the stages 3, 4, and 5 of bud set (e.g. QTLs identified as AAFC 2006 on LGs II, IV, V, and X, see Figures 1-2, 4 and Additional file 5). Assuming moderate heritability values for bud set (e.g. on average 0.43 and 0.36 for cross *P *and *D*, respectively; data not shown), these QTLs explaining 20% of the total phenotypic variance would account for at least 47% of the genetic additive variance. Thus, close to half of genetic variation in bud set may be associated to genomic regions controlled only by declining photoperiod. Given that photoperiod is an environmental factor easily monitorable and stable year after year, genomic regions responding to this factor during bud set could be short-listed for the partial control of height growth *via *the duration of the growing season (see below the section on QTL overlapping). Additional analyses could be conducted to corroborate with accuracy that these bud set QTLs are only associated to photoperiod. A recent study showed that the genes implicated in the photoperiod pathway may be quite conserved among different plant species [98]. Thus, orthologues to the candidate genes of this pathway should be mapped and co-location with bud set QTLs be checked. Though logistically demanding, an additional QTL mapping experiment with long-day and short-day conditions could be undertaken on the large mapping population *D *to obtain a factual measure of photoperiodic response, as done in wheat [99].

For bud flush, QTL clusters identified only from the second cross *D *during the second experimental year, and which were replicated across environments (i.e. QTLs identified as AAFC 2007 or VES 2007 on LGs III, V, and XI, see Figrues 1-2, 4 and Additional file 4), possibly indicate genomic regions responding to temperature variations of the previous winter, or could be linked to the declining photoperiod of the previous summer season. The main factor initiating bud flush is reportedly temperature [100,101]. However, because bud set is associated to the formation of needle primordia through the end of the growing season and declining photoperiod, the height growth for the following year is thus predetermined [102,103]. Therefore, QTLs for bud flush and height growth that were observed only for the second year in both environmental conditions could be closely tied to the declining photoperiod of the previous year via bud set and the formation of needle primordia.

### QTL overlapping among traits

A number of significant occurrences of overlapping QTLs among bud flush, bud set, and height growth were observed after permutation tests among all QTL positions. For instance, on LG X, the confidence intervals of height growth QTLs significantly overlapped with those detected for bud flush and bud set (Figure 4). Another instance of significant co-locations of QTLs was identified for the three adaptive traits on linkage groups III, IV, and V (Figures 1-2). Such co-locations indicate that the shared QTL clusters may bear pleiotrophic effects.

The co-locations of QTLs identified for both phenological traits and height growth in the present study indicated a similar number of overlapping QTL clusters between height growth and bud flush, and between height growth and bud set. In juvenile eastern white spruce, early studies have shown for instance that height growth is correlated to bud set and the length of the growth season, which itself is partly determined by the timing of bud set [32]. While QTLs for bud flush and bud set corresponded to composite phenotypes in our study, correlations between the principal coordinates derived from PCA and the original stages (expressed in Julian days, see Mat&Meth) were also high (data not shown). Thus, establishing the correspondence between QTL co-locations and correlations between phenotypic characters appears possible. Considering together the three adaptive traits studied herein, significant correlations were observed: up to -0.55 between height growth and bud flush, and up to 0.44 between height growth and bud set. These correlations are in the same range and signs as previous estimates in juvenile black spruce [104] and eastern white spruce [32], indicating the opposite effects of these characters on the length of the growth season and ensuing height growth [32]. In different Angiosperms, similar observations could be made where some of the QTLs detected for spring flush, stem height, basal area, and sylleptic branch number in poplar, for bud flush and height growth in oak, or for bunch number and bunch weight in palms, were overlapping on the same linkage groups and could be related to significant correlations among these traits [41,45,77]. Previous quantitative trait genetic studies suggested that trait correlations may be attributable to either pleiotropic effects of single genes or to tight linkage of several genes that individually influence specific traits [105,106]. It should not be too difficult to disentangle these two effects. A few examples of potential quantitative trait nucleotides (QTNs) or SNPs closely link to QTNs would suggest that pleitropy might be involved. For instance, on LG X, the gene SNP *5437a *(e.i. cluster #GQ03507_B21 in Arborea Gene Catalog GCAT version 3.3, ftp://ftp.gydle.com/pub/arborea) was observed as having the highest LOD value simultaneously in QTLs for bud flush, bud set, and height growth. The corresponding gene for this SNP was observed as deregulated (up-expressed) in an expression profile study recently conducted on growth cessation and bud formation in white spruce [107]. On the same linkage group, another gene SNP, e.i. *4148e *(cluster #GQ0203_O13 in Arborea Gene Catalog GCAT version 3.3, ftp://ftp.gydle.com/pub/arborea), was observed as simultaneously associated to bud set and height growth in -1LOD interval under the maximal LOD value of QTLs, so in a highly significant confidence interval. This latter SNP (*4148e*) was identified as candidate for adaptation from a genome scan aimed at finding SNPs with significant differentiation among natural populations of white spruce [108]. If such SNPs influence bud flush or bud set timings, then they would also modulate height growth and could be considered as putative causal SNPs or QTNs (quantitative trait nucleotides). Association studies in unstructured populations should allow to verify these hypotheses. However, even if the same genes or sets of genes are involved in QTL co-locations or overlaps, there should likely be variations in magnitude of effects, as recently observed for common gene sets underlying the developmental control of male and female flowers in maize [106]. Indeed, when orthologous QTLs underlie different traits, the contribution of each of the orthologous genes to the traits might vary. Moreover, a variable part of the covariance between phenotypic characters can also be accounted for by QTLs mapping to different regions of the genome [109].

### QTLs and gene expression profiles

To follow upon the above observations of SNPs possibly tightly linked to QTNs or representing QTNs, we investigated expression profile studies conducted in spruce species in an effort to find genes that would co-localize with QTLs. While there may be several potential candidate genes that mapped within the confidence limits of QTL location, and because only a fraction of the gene space was mapped, care must be taken not to blindly accept the first suitable candidate with a plausible function. Analyses of comprehensive microarray gene expression profiling in white spruce and Sitka spruce [110] have shed light on changes of gene expression during bud formation, growth cessation, and cold acclimation, potentially revealing regulating genes. Several genes differentially expressed during bud development and cold hardiness [110] were found to co-localize with QTLs specifically associated to bud set in the present study. As mentioned in the previous section, the gene SNP *4148e *is a good example. Another possible case is the *BAS1/CyP734A *gene (targeted by the gene SNP *5068f*, i.e. cluster #GQ04107_J01 in Arborea's Gene Catalog GCAT version 3.3, ftp://ftp.gydle.com/pub/arborea), which was included in a QTL for bud set positioned onto LG VIII. This gene was also found to be involved in response to light stimulus and it was up-expressed by short day induction [107]. The *BAS1/CyP734A *gene likely belongs to the brassinosteroid family, which was shown to regulate dormancy [111,112]. Another gene associated to a bud set QTL detected onto LG VI (with the gene SNP *10583v1*, i.e. cluster #GQ03808_I16 in Arborea Gene Catalog GCAT version 3.3, ftp://ftp.gydle.com/pub/arborea) was from the dehydrin family and turned out to be up-expressed after an induction of short days [107]. A SNP of this gene was also previously identified as candidate for adaptation from a genome scan aimed at finding SNPs with significant differentiation among natural populations of white spruce [108]. Its putative role as antifreeze gene may be to stabilize cellular membrane [110]. The up-regulation of this gene after an induction of seven short days and throughout all bud stages until the dormancy [107], suggests that a high level of freezing tolerance could be rapidly achieved, that could decrease frost damages during the timing of bud set.

These few examples, though not comprehensive, indicate that QTL analysis together with gene mapping, population structure studies, and expression profiling can reveal candidate genes for bud formation and growth cessation, suggesting valuable new paths of investigations at the functional level. Many other genes differentially expressed during bud development could also be associated to overlapping bud set and bud flush QTLs, suggesting that these genes could also involved in the biochemical pathways underlying the expression of both traits. However, while bridging the gap between structural, population, and functional genomics and providing new research hypotheses, these temptative associations should not be interpreted as definite proofs of the causal role of the genes identified in the underlying QTLs, because QTL mapping implicates large arrays of genes by simple virtue of linkage.

## Conclusions

Some years ago, Chen et al. [10] suggested that the candidate gene approach would probably become the method of choice for identifying QTLs in most organisms, including forest trees. The present study is a significant effort in this direction. Robust QTL mapping with gene-based linkage maps resulted in a much improved estimation of the genetic architecture of a conifer genome in terms of the magnitude of QTL effects, QTL-environment interactions, and putative pleiotropy. The co-location of candidate genes and QTL intervals cannot to be considered as a strict evidence of these genes being causally involved in the quantitative trait. However, the use of gene maps in conjunction with QTL architecture, gene expression and outlier detection studies also allowed to propose genes and gene SNPs with potential causal roles in the underlying QTLs. Supplementary validations involving large surveys in natural populations and formal association genetic studies [31,108,113], as well as expression studies and functional characterization [114], appear essential to attest such associations [3]. Recent association mapping studies have proven useful to reveal significant associations between genes previously identified in cold-hardiness QTLs [29] and SNPs genotyped in Douglas fir association populations [113], and between gene SNPs putatively involved in light signal transduction and bud set timing variation in Sitka spruce [31].

The next challenge should be to enhance the representation of gene space on genomic maps and narrow down the intervals to small regions that would include small numbers of candidate genes. Such gene maps should also be highly useful for comparative genomics and to orientate and support efforts in sequencing the conifer genome. To instigate this highly challenging task, different strategies should be put forward. Resequencing of expressed sequenced tags (ESTs) using pyrosequencing should allow to identify segregating SNPs and map all expressed genes at a reasonable cost, which currently add up to nearly 28,000 genes in white spruce (Arborea GCAT3.3 gene catalogue). Currently half of the expressed genes is being mapped in white spruce using such strategies and high-throughput genotyping and mapping populations in the order of thousands of progeny. Plans for similar maps are being developed in other conifers, which will facilitate large-scale comparative analysis of genome structure and quantitative traits architecture. These maps should also be useful to prioritize the sequencing of gene-containing BACs corresponding to QTLs or gene-rich regions. Shotgun sequencing of these BACs should represent a useful starting point to conifer genome sequencing, given the mapping of the entire gene space which should facilitate the assembly of gene-rich regions.

A complete understanding of the genes and gene networks underlying traits involved in the control of height growth and adaptive traits associated to bud phenology is crucial, to help orientate association genomics efforts and hasten the development of genomics-assisted selection aimed at increasing forest productivity in the face of an uncertain and rapidly changing environment [18]. Selected genotypes should combine an optimum duration of seasonal height growth with frost hardiness, implicating that timings of bud flush and bud set take place in time to avoid injuries by late frosts in the spring and by early frosts in the fall [93,115-118]. These requirements are expected to change dramatically under northern latitudes, where climate warming has already been shown to be proportionally greater [119].

In a near future, using one of the present mapping populations, a QTL mapping study will integrate the expression profiles of a large number of white spruce genes or expression QTLs (eQTLs). Transcript abundance may act as intermediate phenotype between loci and macroscopic phenotypes, and can be considered as expression quantitative trait (e-trait) in order to identify chromosomal regions where genotypes significantly affect gene expression [120]. By using *cis*- and *trans*- mapping approaches, other interesting questions regarding gene expression regulation could be addressed by combining QTL and eQTL: for instance the relative contributions of *cis*-regulatory elements versus *trans*-regulatory elements [121], or the exploration of the effect of gene duplications on the genetic regulatory network [122]. Because of the virtually unlimited types of data that can be integrated in QTL mapping for an "overall genomic information system" (e.g., eQTL, proteomics, metabolomics, association studies), the increase of gene mapping efforts in conifer species shall represent an important stage for conifer comparative genomics, simultaneously opening stimulating perspectives for evolutionary studies and molecular breeding applications.

## Authors' contributions

BP produced all individual, sub-composite and composite linkage maps, co-performed all statistical analyses of phenotypic traits and principal component analyses of phenological data, constructed linkage maps, tested and conducted various QTL approaches with MapQTL and MultiQTL, evaluated QTL co-locations among traits, compiled and analysed all results, and drafted the manuscript, figures, tables, and additional files. JB co-conceived the study and co-obtained the funding, supervised the gene sequencing, SNP discovery and the design of SNP arrays for white spruce (Arborea PgLM0 and PgLM1 arrays), provided overall guidance during QTL analyses, co-designed and edited the manuscript. PM co-performed all statistical analyses of phenotypic traits and principal component analyses of phenological data and designed a software for QTL visualization. KR supervised the GoldenGate assay developed for white spruce and Sitka spruce (Treenomix project), developed permutation programs to test the co-location of QTLs, and edited the manuscript. NI co-conceived the study and co-obtained the funding, supervised phenotypic data collection, provided the main guidance during linkage mapping and QTL analyses, and co-designed and edited the manuscript. All authors read and approved the final manuscript.

## Supplementary Material

Additional file 1 **List of gene markers retained for linkage map construction**. List of primer sequences used for PCR amplification for each gene-marker retained for the construction of individual linkage maps for both crosses *P *and *D*. Click here for file

Additional file 2 **Measurements of bud flush, bud set and height growth, over three years, two sites and for two unrelated mapping populations**. Statistical parameters of phenotypic data for bud flush, bud set and height growth, over three years, two sites and for two unrelated mapping populations. Click here for file

Additional file 3 **Pearson's and Spearman's (grey background) correlation coefficients for each trait measured in outdoor (VES) and indoor (AAFC) conditions for both mapping populations P and D**. (* significant at 5% level; ** at 1% level; *** at 0.1% level) Phenotypic correlation coefficients within and among the three adaptive traits. Click here for file

Additional file 4 **QTLs identified for bud flush**. List of QTLs identified for bud flush within each mapping population, *P *and *D*, for each environmental condition and year (QTL interval position, PPVE percent of phenotypic variance explained, LOD value, list of gene loci for each QTL). Click here for file

Additional file 5 **QTLs identified for bud set**. List of QTLs identified for bud set within each mapping population, *P *and *D*, for each environmental condition and year (QTL interval position, PPVE percent of phenotypic variance explained, LOD value, list of gene loci for each QTL). Click here for file

Additional file 6 **QTLs identified for height growth**. List of QTLs identified for growth within each mapping population, *P *and *D*, for each environmental condition and year (QTL interval position, PPVE percent of phenotypic variance explained, LOD value, list of gene loci for each QTL). Click here for file
