# Supplementary material for: QTL mapping in white spruce: gene maps and genomic regions underlying adaptive traits across pedigrees, years and environments
Source: BMC Genomics. 2011 Mar 10;12:145. doi: 10.1186/1471-2164-12-145 (PMC3068112; doi:10.1186/1471-2164-12-145)
Supplement: Additional file 2 — Measurements of bud flush, bud set and height growth, over three years, two sites and for two unrelated mapping populations. Statistical parameters of phenotypic data for bud flush, bud set and height growth, over three years, two sites and for two unrelated mapping populations. [file 1471-2164-12-145-S2.DOC]

**Additional file 2.** Measurements of bud flush, bud set and height growth, over three years, two sites and for two unrelated mapping populations.

| Traits and sites ^a^ | Mapping population ^b^ | Year |  | Mean | Standard deviation | Minimum | Quartile 1 | Quartile 3 | Maximum |
| --- | --- | --- | --- | --- | --- | --- | --- | --- | --- |
|  |  |  |  |  |  |  |  |  |  |
| Bud flush (in julian days) | | |  |  |  |  |  |  |  |
| VES | *P* | 2004 |  | 159.0 | 2.3 | 149 | 158 | 160 | 163 |
|  |  | 2005 |  | 152.4 | 2.6 | 146 | 151 | 154 | 159 |
|  |  |  |  |  |  |  |  |  |  |
|  | *D* | 2006 |  | 151.5 | 1.9 | 146 | 150 | 152 | 157 |
|  |  | 2007 |  | 147.6 | 3.0 | 139 | 145 | 149 | 159 |
|  |  |  |  |  |  |  |  |  |  |
| AAFC | *P* | 2004 |  | - | - | - | - | - | - |
|  |  | 2005 |  | - | - | - | - | - | - |
|  |  |  |  |  |  |  |  |  |  |
|  | *D* | 2006 |  | 151.2 | 2.4 | 144 | 149 | 152 | 159 |
|  |  | 2007 |  | 141.0 | 4.0 | 132 | 138 | 144 | 154 |
|  |  |  |  |  |  |  |  |  |  |
| Bud set (in julian days) | | |  |  |  |  |  |  |  |
| VES | *P* | 2004 |  | 223.0 | 9.9 | 202 | 216 | 230 | 253 |
|  |  | 2005 |  | 231.6 | 17.6 | 204 | 216 | 243 | 278 |
|  |  |  |  |  |  |  |  |  |  |
|  | *D* | 2006 |  | 217.2 | 9.9 | 193 | 211 | 224 | 234 |
|  |  | 2007 |  | 203.7 | 10.5 | 187 | 197 | 209 | 268 |
|  |  |  |  |  |  |  |  |  |  |
| AAFC | *P* | 2004 |  | - | - | - | - | - | - |
|  |  | 2005 |  | 209.7 | 7.3 | 201 | 205 | 212 | 273 |
|  |  |  |  |  |  |  |  |  |  |
|  | *D* | 2006 |  | 213.8 | 10.9 | 198 | 205 | 220 | 249 |
|  |  | 2007 |  | 203.0 | 16.3 | 185 | 198 | 204 | 270 |
|  |  |  |  |  |  |  |  |  |  |
| Annual height growth (mm) | | |  |  |  |  |  |  |  |
| VES | *P* | 2004 |  | 54.1 | 13.0 | 22 | 45 | 62 | 113 |
|  |  | 2005 |  | 86.4 | 24.8 | 22 | 70 | 101 | 185 |
|  |  |  |  |  |  |  |  |  |  |
|  | *D* | 2005 |  | 193.9 | 34.1 | 91 | 172 | 217 | 287 |
|  |  | 2006 |  | 120.7 | 33.4 | 43 | 96 | 140 | 237 |
|  |  | 2007 |  | 201.8 | 59.4 | 35 | 163 | 239 | 405 |
|  |  |  |  |  |  |  |  |  |  |
| AAFC | *P* | 2004 |  | - | - | - | - | - | - |
|  |  | 2005 |  | 144.0 | 25.7 | 78 | 127 | 160 | 240 |
|  |  |  |  |  |  |  |  |  |  |
|  | *D* | 2005 |  | 197.1 | 35.4 | 84 | 174 | 221 | 303 |
|  |  | 2006 |  | 109.5 | 29.2 | 33 | 90 | 127 | 206 |
|  |  | 2007 |  | 178.0 | 29.9 | 87 | 158 | 195 | 338 |
|  |  |  |  |  |  |  |  |  |  |
| Total height growth (mm) | | |  |  |  |  |  |  |  |
| VES | *P* | 2006 |  | 477.8 | 61.9 | 285 | 434 | 518 | 655 |
|  |  |  |  |  |  |  |  |  |  |
| AAFC | *D* | 2006 |  | 480.2 | 61.8 | 252 | 439 | 523 | 678 |
|  |  |  |  |  |  |  |  |  |  |
|  |  |  |  |  |  |  |  |  |  |

^a^ AAFC, indoor controlled conditions at Agriculture and Agri-Food Canada ; VES, natural outdoor conditions at Valcartier Experimental Station

^b^ *P*, first mapping population (cross C96-1-2856); *D*, second mapping population (cross C94-1-2516)
