# Supplementary material for: QTL mapping in white spruce: gene maps and genomic regions underlying adaptive traits across pedigrees, years and environments
Source: BMC Genomics. 2011 Mar 10;12:145. doi: 10.1186/1471-2164-12-145 (PMC3068112; doi:10.1186/1471-2164-12-145)
Supplement: Additional file 3 — Pearson's and Spearman's (grey background) correlation coefficients for each trait measured in outdoor (VES) and indoor (AAFC) conditions for both mapping populations P and D. (* significant at 5% level; ** at 1% level; *** at 0.1% level) Phenotypic correlation coefficients within and among the three adaptive traits. [file 1471-2164-12-145-S3.DOC]

**Additional file 3.** Pearson’s and Spearman’s (grey background) correlation coefficients for each trait measured in outdoor (VES) and indoor (AAFC) conditions for both mapping populations *P* and *D*. (* significant at 5% level; ** at 1% level; *** at 0.1% level)

| Cross |  |  |  |  | *P* | |  |  |  |  |  |  |  |  |  |  |  |  |  |  |  |  |  |  |  |  |  |  |
| --- | --- | --- | --- | --- | --- | --- | --- | --- | --- | --- | --- | --- | --- | --- | --- | --- | --- | --- | --- | --- | --- | --- | --- | --- | --- | --- | --- | --- |
| Site |  |  |  |  | **VES** |  |  |  |  |  |  |  |  |  |  |  |  |  |  |  |  |  |  | **AAFC** |  |  |  |  |
|  | Year |  |  |  | 2004 |  |  |  |  |  |  |  |  |  | 2005 |  |  |  |  |  |  |  |  | 2005 |  |  |  |  |
|  | | Trait |  |  | Bud flush | |  |  |  |  | Growth | Bud set |  |  | Bud flush | |  |  |  |  | Growth | Bud set |  | Bud set |  |  |  |  |
|  | |  | Stage |  | stage 1 | stage 2 | stage 3 | stage 4 | stage 5 | stage 6 | annual | stage 1 | stage 3 | stage 5 | stage 1 | stage 2 | stage 3 | stage 4 | stage 5 | stage 6 | annual | stage 1 | stage 3 | stage 1 | stage 3 | stage 5 |  |  |
| *P* | |  |  |  |  |  |  |  |  |  |  |  |  |  |  |  |  |  |  |  |  |  |  |  |  |  |  |  |
| VES |  |  |  |  |  |  |  |  |  |  |  |  |  |  |  |  |  |  |  |  |  |  |  |  |  |  |  |  |
|  | 2004 | Bud flush | 2 |  | 0.583*** |  |  |  |  |  |  |  |  |  |  |  |  |  |  |  |  |  |  |  |  |  |  |  |
|  |  |  | 3 |  | 0.552*** | 0.788*** |  |  |  |  |  |  |  |  |  |  |  |  |  |  |  |  |  |  |  |  |  |  |
|  |  |  | 4 |  | 0.415*** | 0.449*** | 0.777*** |  |  |  |  |  |  |  |  |  |  |  |  |  |  |  |  |  |  |  |  |  |
|  |  |  | 5 |  | 0.453*** | 0.622*** | 0.605*** | 0.550*** |  |  |  |  |  |  |  |  |  |  |  |  |  |  |  |  |  |  |  |  |
|  |  |  | 6 |  | 0.480*** | 0.672*** | 0.644*** | 0.546*** | 0.880*** |  |  |  |  |  |  |  |  |  |  |  |  |  |  |  |  |  |  |  |
|  |  |  |  |  |  |  |  |  |  |  |  |  |  |  |  |  |  |  |  |  |  |  |  |  |  |  |  |  |
|  |  | Growth | annual |  | -0.068 | -0.159** | -0.167** | -0.093 | -0.108 | -0.104 |  |  |  |  |  |  |  |  |  |  |  |  |  |  |  |  |  |  |
|  |  |  |  |  |  |  |  |  |  |  |  |  |  |  |  |  |  |  |  |  |  |  |  |  |  |  |  |  |
|  |  | Bud set | 1 |  | -0.017 | -0.131 | -0.087 | -0.122 | -0.205** | -0.184* | 0.014 |  |  |  |  |  |  |  |  |  |  |  |  |  |  |  |  |  |
|  |  |  | 3 |  | -0.202** | -0.151* | -0.207** | -0.232*** | -0.236*** | -0.226*** | 0.135* | 0.232** |  |  |  |  |  |  |  |  |  |  |  |  |  |  |  |  |
|  |  |  | 5 |  | -0.007 | -0.062 | -0.067 | -0.124* | -0.045 | -0.056 | 0.110 | 0.063 | 0.434*** |  |  |  |  |  |  |  |  |  |  |  |  |  |  |  |
|  |  |  |  |  |  |  |  |  |  |  |  |  |  |  |  |  |  |  |  |  |  |  |  |  |  |  |  |  |
|  | 2005 | Bud flush | 1 |  | 0.043 | -0.041 | -0.132* | -0.083 | -0.066 | -0.059 | - | - | - | - |  |  |  |  |  |  |  |  |  |  |  |  |  |  |
|  |  |  | 2 |  | 0.131* | 0.101 | 0.044 | 0.085 | 0.112 | 0.110 | - | - | - | - | 0.332*** |  |  |  |  |  |  |  |  |  |  |  |  |  |
|  |  |  | 3 |  | 0.063 | 0.078 | 0.051 | 0.106 | 0.122* | 0.125* | - | - | - | - | 0.296*** | 0.510*** |  |  |  |  |  |  |  |  |  |  |  |  |
|  |  |  | 4 |  | 0.017 | 0.005 | 0.045 | 0.113 | 0.117 | 0.110 | - | - | - | - | 0.294*** | 0.408*** | 0.578*** |  |  |  |  |  |  |  |  |  |  |  |
|  |  |  | 5 |  | 0.058 | 0.049 | 0.033 | 0.079 | 0.124* | 0.125* | - | - | - | - | 0.176** | 0.357*** | 0.455*** | 0.640*** |  |  |  |  |  |  |  |  |  |  |
|  |  |  | 6 |  | 0.177** | 0.170** | 0.135* | 0.148* | 0.217*** | 0.264*** | - | - | - | - | 0.181** | 0.282*** | 0.391*** | 0.529*** | 0.658*** |  |  |  |  |  |  |  |  |  |
|  |  |  |  |  |  |  |  |  |  |  |  |  |  |  |  |  |  |  |  |  |  |  |  |  |  |  |  |  |
|  |  | Growth | annual |  | - | - | - | - | - | - | 0.424*** | - | - | - | -0.069 | -0.221*** | -0.087 | -0.040 | -0.108 | -0.043 |  |  |  |  |  |  |  |  |
|  |  |  |  |  |  |  |  |  |  |  |  |  |  |  |  |  |  |  |  |  |  |  |  |  |  |  |  |  |
|  |  | Bud set | 1 |  | - | - | - | - | - | - | - | -0.021 | 0.192** | 0.131* | -0.072 | 0.044 | -0.129 | -0.018 | 0.016 | -0.059 | 0.242*** |  |  |  |  |  |  |  |
|  |  |  | 3 |  | - | - | - | - | - | - | - | 0.071 | 0.192*** | 0.111 | 0.041 | 0.063 | 0.039 | 0.116 | 0.073 | 0.001 | 0.297*** | 0.500*** |  |  |  |  |  |  |
|  |  |  | 5 |  | - | - | - | - | - | - | - | 0.079 | 0.228*** | 0.172** | -0.027 | -0.018 | 0.064 | 0.118 | 0.040 | -0.033 | 0.358*** | 0.277*** | 0.505*** | - | - | - |  |  |
|  |  |  |  |  |  |  |  |  |  |  |  |  |  |  |  |  |  |  |  |  |  |  |  |  |  |  |  |  |
| AAFC | 2005 | Bud set | 1 |  | - | - | - | - | - | - | - | - | - | - | - | - | - | - | - | - | - | - | - | - | - | - |  |  |
|  |  |  | 3 |  | - | - | - | - | - | - | - | - | - | - | - | - | - | - | - | - | - | - | - | -0.155* | - | - |  |  |
|  |  |  | 5 |  | - | - | - | - | - | - | - | - | - | - | - | - | - | - | - | - | - | - | - | -0.077 | 0.322*** | - |  |  |
|  |  |  |  |  |  |  |  |  |  |  |  |  |  |  |  |  |  |  |  |  |  |  |  |  |  |  |  |  |
|  |  | Growth | annual |  | - | - | - | - | - | - | - | - | - | - | - | - | - | - | - | - | - | - | - | -0.003 | 0.331*** | 0.119* |  |  |
|  |  |  |  |  |  |  |  |  |  |  |  |  |  |  |  |  |  |  |  |  |  |  |  |  |  |  |  |  |
|  |  |  |  |  |  |  |  |  |  |  |  |  |  |  |  |  |  |  |  |  |  |  |  |  |  |  |  |  |
|  |  |  |  |  |  |  |  |  |  |  |  |  |  |  |  |  |  |  |  |  |  |  |  |  |  |  |  |  |
|  |  |  |  |  |  |  |  |  |  |  |  |  |  |  |  |  |  |  |  |  |  |  |  |  |  |  |  |  |
| Additional file 3 (continued) | | | | | | |  |  |  |  |  |  |  |  |  |  |  |  |  |  |  |  |  |  |  |  |  |  |
| Cross |  |  |  |  | *D* | |  |  |  |  |  |  |  |  |  |  |  |  |  |  |  |  |  |  |  |  |  |  |
| **Site** |  |  |  |  | VES |  |  |  |  |  |  |  |  |  |  |  |  |  |  |  |  |  |  |  |  |  |  |  |
|  | Year |  |  |  | 2005 | 2006 |  |  |  |  |  |  |  |  |  |  |  | 2007 |  |  |  |  |  |  |  |  |  |  |
|  |  | Trait |  |  | Growth | Bud flush | |  |  |  |  | Growth | Bud set |  |  |  |  | Bud flush | |  |  |  |  | Growth | Bud set |  |  |  |
|  |  |  | Stage |  |  | stage 1 | stage 2 | stage 3 | stage 4 | stage 5 | stage 6 | annual | stage 2 | stage 3 | stage 4 | stage 5 |  | stage 1 | stage 2 | stage 3 | stage 4 | stage 5 | stage 6 | annual | stage 2 | stage 3 | stage 4 |  |
| *D* | |  |  |  |  |  |  |  |  |  |  |  |  |  |  |  |  |  |  |  |  |  |  |  |  |  |  |  |
| VES |  |  |  |  |  |  |  |  |  |  |  |  |  |  |  |  |  |  |  |  |  |  |  |  |  |  |  |  |
|  | 2006 | Bud flush | 1 |  | - |  |  |  |  |  |  |  |  |  |  |  |  |  |  |  |  |  |  |  |  |  |  |  |
|  |  |  | 2 |  | - | 0.690*** |  |  |  |  |  |  |  |  |  |  |  |  |  |  |  |  |  |  |  |  |  |  |
|  |  |  | 3 |  | - | 0.545*** | 0.701*** |  |  |  |  |  |  |  |  |  |  |  |  |  |  |  |  |  |  |  |  |  |
|  |  |  | 4 |  | - | 0.372*** | 0.530*** | 0.612*** |  |  |  |  |  |  |  |  |  |  |  |  |  |  |  |  |  |  |  |  |
|  |  |  | 5 |  | - | 0.290*** | 0.497*** | 0.544*** | 0.634*** |  |  |  |  |  |  |  |  |  |  |  |  |  |  |  |  |  |  |  |
|  |  |  | 6 |  | - | 0.263*** | 0.474*** | 0.531*** | 0.593*** | 0.757*** |  |  |  |  |  |  |  |  |  |  |  |  |  |  |  |  |  |  |
|  |  |  |  |  |  |  |  |  |  |  |  |  |  |  |  |  |  |  |  |  |  |  |  |  |  |  |  |  |
|  |  | Growth | annual |  | 0.134*** | -0.312*** | -0.368*** | -0.325*** | -0.229*** | -0.179*** | -0.212*** |  |  |  |  |  |  |  |  |  |  |  |  |  |  |  |  |  |
|  |  |  |  |  |  |  |  |  |  |  |  |  |  |  |  |  |  |  |  |  |  |  |  |  |  |  |  |  |
|  |  | Bud set | 2 |  | - | 0.002 | -0.056 | -0.067 | 0.012 | -0.099 | -0.015 | -0.053 |  |  |  |  |  |  |  |  |  |  |  |  |  |  |  |  |
|  |  |  | 3 |  | - | 0.006 | -0.047 | 0.047 | -0.023 | -0.057 | -0.021 | 0.101* | 0.301*** |  |  |  |  |  |  |  |  |  |  |  |  |  |  |  |
|  |  |  | 4 |  | - | 0.010 | 0.057 | 0.025 | 0.003 | 0.059 | 0.065 | 0.072 | 0.085 | 0.392*** |  |  |  |  |  |  |  |  |  |  |  |  |  |  |
|  |  |  | 5 |  | - | -0.075 | -0.046 | -0.017 | 0.042 | 0.054 | -0.007 | 0.060 | 0.049 | 0.074 | 0.333*** |  |  |  |  |  |  |  |  |  |  |  |  |  |
|  |  |  |  |  |  |  |  |  |  |  |  |  |  |  |  |  |  |  |  |  |  |  |  |  |  |  |  |  |
|  |  |  |  |  |  |  |  |  |  |  |  |  |  |  |  |  |  |  |  |  |  |  |  |  |  |  |  |  |
|  | 2007 | Bud flush | 1 |  | - | -0.001 | -0.008 | 0.002 | 0.015 | 0.042 | 0.003 | 0.026 | - | - | - | - |  |  |  |  |  |  |  |  |  |  |  |  |
|  |  |  | 2 |  | - | 0.062 | 0.109* | 0.065 | 0.075 | 0.076 | 0.078 | -0.075 | - | - | - | - |  | 0.189*** |  |  |  |  |  |  |  |  |  |  |
|  |  |  | 3 |  | - | 0.152*** | 0.148*** | 0.102* | 0.096* | 0.064 | 0.018 | 0.086 | - | - | - | - |  | 0.143** | 0.357*** |  |  |  |  |  |  |  |  |  |
|  |  |  | 4 |  | - | 0.148*** | 0.135** | 0.068 | 0.063 | 0.126** | 0.109* | 0.132** | - | - | - | - |  | 0.139** | 0.289*** | 0.591*** |  |  |  |  |  |  |  |  |
|  |  |  | 5 |  | - | 0.081 | 0.059 | 0.026 | 0.039 | 0.123** | 0.074 | 0.087 | - | - | - | - |  | -0.007 | 0.274*** | 0.415*** | 0.637*** |  |  |  |  |  |  |  |
|  |  |  | 6 |  | - | 0.038 | 0.019 | -0.001 | 0.017 | 0.129** | 0.098* | 0.152*** | - | - | - | - |  | 0.047 | 0.253*** | 0.380*** | 0.614*** | 0.784*** |  |  |  |  |  |  |
|  |  |  |  |  |  |  |  |  |  |  |  |  |  |  |  |  |  |  |  |  |  |  |  |  |  |  |  |  |
|  |  | Growth | annual |  | 0.135*** | -0.062 | -0.085 | -0.037 | -0.034 | 0.057 | 0.093* | 0.149*** | - | - | - | - |  | -0.032 | -0.187*** | -0.345*** | -0.195*** | -0.077 | -0.050 |  |  |  |  |  |
|  |  |  |  |  |  |  |  |  |  |  |  |  |  |  |  |  |  |  |  |  |  |  |  |  |  |  |  |  |
|  |  | Bud set | 2 |  | - | - | - | - | - | - | - | - | 0.060 | 0.160** | 0.025 | 0.016 |  | -0.053 | -0.058 | -0.091 | 0.006 | 0.128** | 0.189*** | 0.303*** |  |  |  |  |
|  |  |  | 3 |  | - | - | - | - | - | - | - | - | 0.046 | 0.147** | 0.077 | 0.060 |  | -0.066 | -0.097* | -0.133** | -0.001 | 0.095 | 0.160*** | 0.378*** | 0.751*** |  |  |  |
|  |  |  | 4 |  | - | - | - | - | - | - | - | - | -0.058 | 0.174*** | 0.134* | 0.034 |  | 0.030 | -0.062 | -0.058 | 0.059 | 0.157** | 0.180*** | 0.367*** | 0.629*** | 0.725*** |  |  |
|  |  |  | 5 |  | - | - | - | - | - | - | - | - | 0.007 | 0.163** | 0.236*** | 0.147* |  | -0.039 | -0.084 | -0.071 | 0.020 | 0.106 | 0.116* | 0.437*** | 0.480*** | 0.608*** | 0.835*** |  |
|  |  |  |  |  |  |  |  |  |  |  |  |  |  |  |  |  |  |  |  |  |  |  |  |  |  |  |  |  |
|  |  |  |  |  |  |  |  |  |  |  |  |  |  |  |  |  |  |  |  |  |  |  |  |  |  |  |  |  |
|  |  |  |  | |  |  |  |  |  |  |  |  |  |  |  |  |  |  |  |  |  |  |  |  |  |  |  |  |
|  |  |  |  | |  |  |  |  |  |  |  |  |  |  |  |  |  |  |  |  |  |  |  |  |  |  |  |  |
|  |  |  |  |  |  |  |  |  |  |  |  |  |  |  |  |  |  |  |  |  |  |  |  |  |  |  |
|  |  |  |  |  |  |  |  |  |  |  |  |  |  |  |  |  |  |  |  |  |  |  |  |  |  |  |
|  |  |  |  |  |  |  |  |  |  |  |  |  |  |  |  |  |  |  |  |  |  |  |  |  |  |  |
|  |  |  |  |  |  |  |  |  |  |  |  |  |  |  |  |  |  |  |  |  |  |  |  |  |  |  |
| Additional file 3 (continued) | | | | | | |  |  |  |  |  |  |  |  |  |  |  |  |  |  |  |  |  |  |  |  |  |  |
| Cross |  |  |  |  | *D* | |  |  |  |  |  |  |  |  |  |  |  |  |  |  |  |  |  |  |  |  |  |  |
| **Site** |  |  |  |  | AAFC |  |  |  |  |  |  |  |  |  |  |  |  |  |  |  |  |  |  |  |  |  |  |  |
|  | Year |  |  |  | 2005 | 2006 |  |  |  |  |  |  |  |  |  |  |  | 2007 |  |  |  |  |  |  |  |  |  |  |
|  |  | Trait |  |  | Growth | Bud flush | |  |  |  |  | Growth | Bud set |  |  |  |  | Bud flush | |  |  |  |  | Growth | Bud set |  |  |  |
|  |  |  | Stage^a^ |  |  | stage 1 | stage 2 | stage 3 | stage 4 | stage 5 | stage 6 | annual | stage 2 | stage 3 | stage 4 | stage 5 |  | stage 1 | stage 2 | stage 3 | stage 4 | stage 5 | stage 6 | annual | stage 1 | stage 2 | stage 3 | stage 4 |
| *D* | |  |  |  |  |  |  |  |  |  |  |  |  |  |  |  |  |  |  |  |  |  |  |  |  |  |  |  |
| **AAFC** |  |  |  |  |  |  |  |  |  |  |  |  |  |  |  |  |  |  |  |  |  |  |  |  |  |  |  |  |
|  | 2006 | Bud flush | 1 |  | - |  |  |  |  |  |  |  |  |  |  |  |  |  |  |  |  |  |  |  |  |  |  |  |
|  |  |  | 2 |  | - | 0.790*** |  |  |  |  |  |  |  |  |  |  |  |  |  |  |  |  |  |  |  |  |  |  |
|  |  |  | 3 |  | - | 0.630*** | 0.760*** |  |  |  |  |  |  |  |  |  |  |  |  |  |  |  |  |  |  |  |  |  |
|  |  |  | 4 |  | - | 0.404*** | 0.549*** | 0.641*** |  |  |  |  |  |  |  |  |  |  |  |  |  |  |  |  |  |  |  |  |
|  |  |  | 5 |  | - | 0.416*** | 0.494*** | 0.564*** | 0.733*** |  |  |  |  |  |  |  |  |  |  |  |  |  |  |  |  |  |  |  |
|  |  |  | 6 |  | - | 0.359*** | 0.470*** | 0.539*** | 0.689*** | 0.873*** |  |  |  |  |  |  |  |  |  |  |  |  |  |  |  |  |  |  |
|  |  |  |  |  |  |  |  |  |  |  |  |  |  |  |  |  |  |  |  |  |  |  |  |  |  |  |  |  |
|  |  | Growth | annual |  | 0.111** | -0.428*** | -0.504*** | -0.546*** | -0.517*** | -0.495*** | -0.493*** |  |  |  |  |  |  |  |  |  |  |  |  |  |  |  |  |  |
|  |  |  |  |  |  |  |  |  |  |  |  |  |  |  |  |  |  |  |  |  |  |  |  |  |  |  |  |  |
|  |  | Bud set | 2 |  | - | 0.105 | 0.039 | 0.063 | 0.059 | 0.036 | 0.054 | -0.134 |  |  |  |  |  |  |  |  |  |  |  |  |  |  |  |  |
|  |  |  | 3 |  | - | 0.053 | 0.015 | 0.056 | 0.023 | 0.013 | 0.013 | 0.026 | 0.338*** |  |  |  |  |  |  |  |  |  |  |  |  |  |  |  |
|  |  |  | 4 |  | - | -0.015 | 0.044 | 0.053 | -0.003 | -0.030 | -0.014 | 0.059 | 0.409*** | 0.449*** |  |  |  |  |  |  |  |  |  |  |  |  |  |  |
|  |  |  | 5 |  | - | -0.038 | -0.030 | -0.018 | -0.073 | -0.049 | -0.047 | 0.080 | 0.399*** | 0.385*** | 0.692*** |  |  |  |  |  |  |  |  |  |  |  |  |  |
|  |  |  |  |  |  |  |  |  |  |  |  |  |  |  |  |  |  |  |  |  |  |  |  |  |  |  |  |  |
|  |  |  |  |  |  |  |  |  |  |  |  |  |  |  |  |  |  |  |  |  |  |  |  |  |  |  |  |  |
|  | 2007 | Bud flush | 1 |  | - | 0.010 | -0.009 | 0.016 | 0.002 | 0.014 | 0.027 | -0.070 | - | - | - | - |  |  |  |  |  |  |  |  |  |  |  |  |
|  |  |  | 2 |  | - | -0.001 | 0.064 | 0.057 | 0.031 | 0.041 | -0.009 | 0.023 | - | - | - | - |  | 0.300*** |  |  |  |  |  |  |  |  |  |  |
|  |  |  | 3 |  | - | 0.075 | 0.037 | 0.083 | 0.011 | 0.044 | -0.009 | -0.015 | - | - | - | - |  | 0.400*** | 0.306*** |  |  |  |  |  |  |  |  |  |
|  |  |  | 4 |  | - | 0.043 | 0.020 | 0.120** | 0.108* | 0.069 | 0.011 | -0.070 | - | - | - | - |  | 0.408*** | 0.321*** | 0.652*** |  |  |  |  |  |  |  |  |
|  |  |  | 5 |  | - | 0.051 | 0.029 | 0.106* | 0.088 | 0.034 | 0.028 | -0.093* | - | - | - | - |  | 0.361*** | 0.379*** | 0.620*** | 0.777*** |  |  |  |  |  |  |  |
|  |  |  | 6 |  | - | 0.022 | 0.021 | 0.118** | 0.126** | 0.074 | 0.041 | -0.055 | - | - | - | - |  | 0.350*** | 0.357*** | 0.588*** | 0.722*** | 0.853*** |  |  |  |  |  |  |
|  |  |  |  |  |  |  |  |  |  |  |  |  |  |  |  |  |  |  |  |  |  |  |  |  |  |  |  |  |
|  |  | Growth | annual |  | 0.324*** | -0.098* | -0.149** | -0.101* | -0.052 | -0.013 | -0.037 | 0.340*** | - | - | - | - |  | -0.187*** | -0.040 | -0.256*** | -0.267*** | -0.321*** | -0.345*** |  |  |  |  |  |
|  |  |  |  |  |  |  |  |  |  |  |  |  |  |  |  |  |  |  |  |  |  |  |  |  |  |  |  |  |
|  |  | Bud set | 1 |  | - | - | - | - | - | - | - | - | -0.020 | 0.078 | -0.037 | -0.043 |  | -0.051 | 0.054 | 0.068 | 0.107* | 0.039 | 0.067 | 0.180*** |  |  |  |  |
|  |  |  | 2 |  | - | - | - | - | - | - | - | - | -0.042 | 0.061 | 0.029 | 0.029 |  | 0.007 | 0.042 | 0.102* | 0.084 | 0.053 | 0.034 | 0.148*** | 0.684*** |  |  |  |
|  |  |  | 3 |  | - | - | - | - | - | - | - | - | -0.102 | 0.072 | 0.040 | 0.049 |  | -0.033 | 0.051 | 0.052 | 0.053 | 0.029 | 0.021 | 0.133*** | 0.616*** | 0.748*** |  |  |
|  |  |  | 4 |  | - | - | - | - | - | - | - | - | -0.107 | 0.033 | 0.060 | -0.004 |  | -0.064 | 0.092 | -0.013 | 0.046 | 0.017 | -0.010 | 0.172*** | 0.598*** | 0.606*** | 0.652*** |  |
|  |  |  | 5 |  | - | - | - | - | - | - | - | - | -0.130 | 0.110 | 0.107 | 0.061 |  | -0.047 | -0.016 | 0.022 | 0.126 | 0.135 | 0.106 | 0.187*** | 0.414*** | 0.431*** | 0.425*** | 0.653*** |
|  |  |  |  |  |  |  |  |  |  |  |  |  |  |  |  |  |  |  |  |  |  |  |  |  |  |  |  |  |

^a^ Pearson correlations were based for each genotyped tree on the average of first day (in julian day) where each stage of bud flush or bud set was reached. Stages of bud flush and bud set are described in Dhont et al. 2010.
