# Supplementary material for: QTL mapping in white spruce: gene maps and genomic regions underlying adaptive traits across pedigrees, years and environments
Source: BMC Genomics. 2011 Mar 10;12:145. doi: 10.1186/1471-2164-12-145 (PMC3068112; doi:10.1186/1471-2164-12-145)
Supplement: Additional file 4 — QTLs identified for bud flush. List of QTLs identified for bud flush within each mapping population, P and D, for each environmental condition and year (QTL interval position, PPVE percent of phenotypic variance explained, LOD value, list of gene loci for each QTL). [file 1471-2164-12-145-S4.DOC]

**Additional file 4**. QTLs identified for bud flush.

|  |  |  |  | Mapping populations | | | | |  | Environmental conditions^c^ |  | Principal component^d^ |  | QTL interval^e^ | | | | | |  | Peak gene | | | | |  | LOD threshold | |
| --- | --- | --- | --- | --- | --- | --- | --- | --- | --- | --- | --- | --- | --- | --- | --- | --- | --- | --- | --- | --- | --- | --- | --- | --- | --- | --- | --- | --- |
| Linkage group^a^ |  | Year^b^ |  | *P*  (C96-1-2856) | |  | *D*  (C94-1-2516) | |  |  |  |  |  | Position at -1LOD |  | Gene loci at -1LOD | |  | PPVE^f^ |  | Position in cM |  | Marker-SNP |  | PPVE^f^ |  | At linkage group level | At genome level |
|  |  |  |  | ♀ 80112 | ♂ 80109 |  | ♀ 77111 | ♂ 2388 |  |  |  |  |  | (-2LOD) |  | (-2LOD) | |  | (LOD max)^g^ |  |  |  |  |  | (LOD)^g^ |  |  |  |
| **III** |  | 2007 |  |  |  |  | 77111 |  |  | Indoor (AAFC) |  | PC1 |  | 74.02-101.29 (68.02-144.31) |  | **12531a, 10460a, sb62, 7398b, 9027f, 6517i, 5488i, 3713a,** (10659-2, 2239a, 10660a, 13661a, 9147b, 2473e, 10759k, 8456j, 14683a, 9889e, 9863a, 14745e) | |  | 3 (2.96 ns) |  | 89.8 |  | 5488j |  | 2.7 (2.94 ns) |  | 3.01 | 4.4 |
|  |  | 2007 |  |  |  |  |  | 2388 |  | Outdoor (VES) |  | PC1 |  | 29.03-47.1 (23.26-53.06) |  | (9572h, 6616m), **5351e,** (12531e) | |  | 9.3 (3.69) |  | 31.1 |  | 5351e |  | 4 (3.01) |  | 2.95 | 4.1 |
|  |  | 2007 |  |  |  |  |  | 2388 |  | Indoor (AAFC) |  | PC1 |  | 38.10-62.51 (31.10-82.97) |  | (5351e) **12531e, 5488i, 9027f, 6517i, sb62f, 10460e, 10129, 7398f,**  (3713d, 10659-2) | |  | 3.6 (3.51) |  | 52.56 |  | 12531e |  | 3.3 (3.41) |  | 3.08 | 6.44 |
|  |  |  |  |  |  |  |  |  |  |  |  |  |  |  |  |  |  |  |  |  |  |  |  |  |  |  |  |  |
| **IV** |  | 2004 |  | 80112 |  |  |  |  |  | Outdoor (VES) |  | PC1 |  | 0.0-19.22 (0.0-26.03) |  | **9780a, 6706p1, 10378,** (3673, Contig-839-121, 16369a) | |  | 7.4 (3.79) |  | 15.07 |  | 10378 |  | 7.4 (3.79) |  | 3.01 | 5.77 |
|  |  | 2005 |  | 80112 |  |  |  |  |  | Outdoor (VES) |  | PC2 |  | 19.94-28.03 (0.0-35.16) |  | (9780a, 6706, 10378, 3673), **Contig-839-121, 16369a** (5775) | |  | 10.9 (3.95) |  | 23.03 |  | 16369a |  | 10.9 (3.95) |  | 2.89 | 4.41 |
|  |  | 2004 |  |  | 80109 |  |  |  |  | Outdoor (VES) |  | PC1 |  | 44.02-71.72 (44.02-84.22) |  | **Contig-605-491, 3056e, 4632, 10378, 6706p1, 12753e,** (3673, 16369a) | |  | 6.2 (3.15) |  | 66.73 |  | 10378 |  | 6.2 (3.15) |  | 3 | 5.44 |
|  |  | 2005 |  |  | 80109 |  |  |  |  | Outdoor (VES) |  | PC2 |  | 67.54-83.22 (49.23-88.78) |  | (Contig-605-491, 3056e, 4632, 10378, 6706p1), **12753e, 3673, 16369a,** (5775) | |  | 10.7 (3.61) |  | 75.96 |  | 16369a |  | 9.9 (3.55) |  | 3.03 | 4.39 |
|  |  | 2007 |  |  |  |  |  | 2388 |  | Indoor (AAFC) |  | PC1 |  | 18.01-48.51 (0-52.51) |  | (b54k, 10193e, 14265a, 7005e, b140m, 10438f, 9693v3, 4045h, 4613k, 650e) **1529e** | |  | 6.8 (3.86) |  | 45.51 |  | 1529e |  | 3.7 (3.39) |  | 2.93 | 6.44 |
|  |  | 2004 |  | 80112 |  |  |  |  |  | Outdoor (VES) |  | PC2 |  | 84.26-102.25 (84.26-111.61) |  | **90012,** (4073) | |  | 8.8 (3.66) |  | 97.8 |  | 90012 |  | 6.9 (3.52) |  | 2.93 | 4.88 |
|  |  | 2004 |  |  | 80109 |  |  |  |  | Outdoor (VES) |  | PC2 |  | 134.56-162.31 (127.56-173.24) |  | **90012,** (4073, Contig-448-294, 8438, Contig-3655-401) | |  | 7.8 (3.37) |  | 154.04 |  | 90012 |  | 6.4 (3.27) |  | 3.18 | 4.56 |
|  |  | 2004 |  |  | 80109 |  |  |  |  | Outdoor (VES) |  | PC1 |  | 162.31-173.24 (140.56-173.24) |  | (90012, 4073), **Contig-448-294, 8438, Contig-3655-401** | |  | 7.1 (3.35) |  | 173.24 |  | Contig-3655-401 |  | 7.1 (3.35) |  | 3 | 5.44 |
|  |  |  |  |  |  |  |  |  |  |  |  |  |  |  |  |  |  |  |  |  |  |  |  |  |  |  |  |  |
| **V** |  | 2007 |  |  |  |  | 77111 |  |  | Indoor (AAFC) |  | PC1 |  | 54.52-61.21 (51.89-65.95) |  | **2510b, 11390a,** (7050a) | |  | 9.6 (5.44) |  | 54.52 |  | **2510b** |  | 9.8 (5.26) |  | 2.88 | 4.4 |
|  |  | 2007 |  |  |  |  |  | 2388 |  | Indoor (AAFC) |  | PC1 |  | 42.50-54.5 (42.50-62.05) |  | **2510f, sb16Mh, 2654e** | |  | 12.2 (6.41) |  | 42.5 |  | **2510f** |  | 12.2 (6.41) |  | 2.89 | 6.44 |
|  |  | 2007 |  |  |  |  |  | 2388 |  | Outdoor (VES) |  | PC2 |  | 21.85-52.05 (14.53-61.05) |  | (7114e), **2510f, sb16M, 2654e** | |  | 3.7 (3.14) |  | 45.28 |  | sb16Mh |  | 3.6 (3.1) |  | 3.02 | 4.44 |
|  |  | 2007 |  |  |  |  | 77111 |  |  | Indoor (AAFC) |  | PC1 |  | 99.45-112.68 (97.45-116.68) |  | **10797-4, 6718e, 6922b** | |  | 5.6 (5.46) |  | 102.68 |  | 6922b |  | 5.1 (5.43) |  | 2.88 | 4.4 |
|  |  | 2007 |  |  |  |  |  | 2388 |  | Indoor (AAFC) |  | PC1 |  | 74.24-84.73 (70.74-89.73) |  | (15170g, 8398m, 6647e), **6718e, 6922a, 7178a, 10049e** | |  | 5.4 (5.66) |  | 78.7 |  | 6922a |  | 5.4 (5.66) |  | 2.89 | 6.44 |
|  |  |  |  |  |  |  |  |  |  |  |  |  |  |  |  |  |  |  |  |  |  |  |  |  |  |  |  |  |
| **VI** |  | 2006 |  |  |  |  | 77111 |  |  | Indoor (AAFC) |  | PC2 |  | 133.03-155.46 (125.03-195.13) |  | **7115c, 11157f, 9524a, 6899a,** (4312c, 4370j, 3053b) | |  | 7.7 (4.68) |  | 151.86 |  | **11157f** |  | 7.7 (4.68) |  | 2.78 | 4.29 |
|  |  | 2006 |  |  |  |  |  | 2388 |  | Outdoor (VES) |  | PC1 |  | 125.96-143.41 (120.26-145.41) |  | **6899e,** (4370e) | |  | 11.6 (10.04) |  | 125.96 |  | **6899e** |  | 12.2 (9.56) |  | 3.12 | 4.22 |
|  |  | 2007 |  |  |  |  | 77111 |  |  | Outdoor (VES) |  | PC1 |  | 165.57-195.13 (133.03-195.13) |  | (7115c, 11157f, 9524a, 6899a, 4312c, 4370j) **3053b** | |  | 9.5 (3.7) |  | 195.13 |  | 3053b |  | 9.8 (3.67) |  | 2.86 | 4.17 |
|  |  | 2006 |  |  |  |  |  | 2388 |  | Indoor (AAFC) |  | PC2 |  | 129.41-152.01 (116.26-152.01) |  | (6899e, 4370e) **1667e, 6769e, 9620t1** | |  | 4.3 (3.29) |  | 152.01 |  | 9620t1 |  | 4.3 (3.29) |  | 3 | 4.5 |
|  |  |  |  |  |  |  |  |  |  |  |  |  |  |  |  |  |  |  |  |  |  |  |  |  |  |  |  |  |
| **X** |  | 2007 |  |  |  |  | 77111 |  |  | Indoor (AAFC) |  | PC1 |  | 99.26-123.98 (99.26-130.97) |  | **7736b, 5437a, 1686c, 2871a** (8228m, 4148e, 5731j) | |  | 3.9 (2.62) |  | 107.06 |  | 5437a |  | 3.9 (2.61) |  | 2.37 | 4.4 |
|  |  | 2007 |  |  |  |  |  | 2388 |  | Indoor (AAFC) |  | PC1 |  | 72.64-83.03 (66.68-94.54) |  | (13366f, 7914h, 7235e), **7736e** (10998e, 4348f) | |  | 3.6 (3.79) |  | 80.39 |  | 7736e |  | 3.5 (3.78) |  | 2.5 | 6.44 |
|  |  |  |  |  |  |  |  |  |  |  |  |  |  |  |  |  |  |  |  |  |  |  |  |  |  |  |  |  |
| **XI** |  | 2005 |  | 80112 |  |  |  |  |  | Outdoor (VES) |  | PC2 |  | 3.90-26.34 (0.0-43.97) |  | (Contig-2685-179), **Contig-126-147, 6202, 7636p2, sb01** (6581) | |  | 16.4 (3.33) |  | 16.3 |  | 7636p2 |  | 16.4 (3.33) |  | 3.17 | 4.41 |
|  |  | 2006 |  |  |  |  | 77111 |  |  | Indoor (AAFC) |  | PC2 |  | 12.44-37.12 (5.21-42.94) |  | (7562c, 7636p2) **6581a, 8739j** | |  | 5.7 (3.22) |  | 25.92 |  | 6581a |  | 4.3 (3.14) |  | 3.02 | 4.29 |
|  |  | 2006 |  |  |  |  |  | 2388 |  | Indoor (AAFC) |  | PC2 |  | 16.34-29.62 (10.53-35.62) |  | (7717f), **6581f, 10300f, 8739j** | |  | 5.5 (3.5) |  | 18.9 |  | 6581f |  | 5.5 (3.5) |  | 3.16 | 4.5 |
|  |  | 2007 |  |  |  |  |  | 2388 |  | Outdoor (VES) |  | PC1 |  | 1.75-21.10 (0.0-80.08) |  | (9861k, 3364v1, 7670e) **10027-3, 5167j, 7636p2, 7562b, 7717f, 6581f, 10300f**, (8739j, sb06f, sb51e, 5290k, 5965e, 3931e, b19g,11945e) | |  | 3.1 (3.01) |  | 8.56 |  | 7636p2 |  | 2.9 (2.95 ns) |  | 2.98 | 4.1 |
|  |  | 2007 |  |  |  |  | 77111 |  |  | Outdoor (VES) |  | PC1 |  | 33.12-50.05 (27.12-52.75) |  | **sb29b, sb06i, 3947e, 1720a, 7849a, 2470e,** (8895b) | |  | 4.4 (4.42) |  | 42.94 |  | **sb29b** |  | 4.4 (4.42) |  | 2.96 | 4.17 |
|  |  | 2007 |  |  |  |  | 77111 |  |  | Outdoor (VES) |  | PC2 |  | 31.12-50.05 (27.12-56.75) |  | **sb29b, sb06i, 3947e, 1720a, 7849a, 2470e,** (8895b, 5290k) | |  | 6.3 (5.53) |  | 45.79 |  | **1720a** |  | 6.3 (5.53) |  | 3.07 | 4.16 |
|  |  | 2007 |  |  |  |  |  | 2388 |  | Indoor (AAFC) |  | PC1 |  | 21.62-53.99 (3.23-63.21) |  | (5167j, 7636p2, 7562b, 7717f, 6581f, 10300f, 8739j), **sb06f** (sb51e) | |  | 3.6 (3.16) |  | 38.99 |  | sb06f |  | 2.8 (3.02) |  | 2.9 | 6.44 |
|  |  | 2007 |  |  |  |  |  | 2388 |  | Outdoor (VES) |  | PC1 |  | 33.62-58.21 (26.62-61.21) |  | **sb06f, sb51e** | |  | 5.8 (4.93) |  | 54.21 |  | **sb51e** |  | 4.9 (4.67) |  | 2.98 | 4.1 |
|  |  | 2007 |  |  |  |  |  | 2388 |  | Outdoor (VES) |  | PC2 |  | 38.62-60.21 (25.62-63.21) |  | **sb06f, sb51e** | |  | 7 (6.01) |  | 54.21 |  | **sb51e** |  | 6.4 (5.89) |  | 2.96 | 4.25 |
|  |  |  |  |  |  |  |  |  |  |  |  |  |  |  |  |  |  |  |  |  |  |  |  |  |  |  |  |  |
| **XII** |  | 2007 |  |  |  |  |  | 2388 |  | Indoor (AAFC) |  | PC1 |  | 5.59-33.64 (0.0-39.71) |  | (7400e, 90001e) **7602a, 6105j, 8339b, 7414p1, 6340e** (9953e) | |  | 3.8 (3.6) |  | 20.07 |  | 8339b |  | 3.3 (3.54) |  | 2.9 | 6.44 |
|  |  | 2007 |  |  |  |  | 77111 |  |  | Indoor (AAFC) |  | PC1 |  | 5.86-26.79 (0.0-35.79) |  | (90001e, 5139v2) **7602a, 9349, 6105e, 7414p1, 6340a** | |  | 3.7 (3.61) |  | 14.45 |  | 6105e |  | 3.7 (3.61) |  | 2.99 | 4.4 |

^a^ Two different colors of green distinguish the individual QTLs associated at two QTL-regions identified on the same linkage group.

^b^ Years of measurements: 2004 and 2005 for the mapping population *P* and, 2006 and 2007 for the mapping population *D*.

^c^ Environmental conditions tested: in a large growth chamber (i.e. indoor conditions) at Agriculture and Agri-Food Canada (AAFC) under a declining photoperiod and day/night-time temperatures of 24/15°C; at Valcartier Experimental Station (VES) under natural outdoor conditions.

^d^ Principal component retained after PCA (see in Materials and Methods; Table 2).

^e^ Confidence interval of each QTL calculated by one-LOD and two-LOD below the QTL-LOD peak (i.e. at -1LOD and -2LOD).

^f^ Proportion of phenotypic variance explained.

^g^ ns = non significant.
