# Supplementary material for: QTL mapping in white spruce: gene maps and genomic regions underlying adaptive traits across pedigrees, years and environments
Source: BMC Genomics. 2011 Mar 10;12:145. doi: 10.1186/1471-2164-12-145 (PMC3068112; doi:10.1186/1471-2164-12-145)
Supplement: Additional file 5 — QTLs identified for bud set. List of QTLs identified for bud set within each mapping population, P and D, for each environmental condition and year (QTL interval position, PPVE percent of phenotypic variance explained, LOD value, list of gene loci for each QTL). [file 1471-2164-12-145-S5.DOC]

**Additional file 5**. QTLs identified for bud set.

|  |  |  |  | Mapping populations | | | | |  | Environmental conditions^c^ |  | Principal component^d^ |  | QTL interval^e^ | | | | |  | Peak gene | | | | |  | LOD threshold | |
| --- | --- | --- | --- | --- | --- | --- | --- | --- | --- | --- | --- | --- | --- | --- | --- | --- | --- | --- | --- | --- | --- | --- | --- | --- | --- | --- | --- |
| Linkage group^a^ |  | Year^b^ |  | *P*  (C96-1-2856) | |  | *D*  (C94-1-2516) | |  |  |  |  |  | Position at -1LOD |  | Gene loci at -1LOD |  | PPVE^f^ |  | Position in cM |  | Marker-SNP |  | PPVE^f^ |  | At linkage group level | At genome level |
|  |  |  |  | ♀ 80112 | ♂ 80109 |  | ♀ 77111 | ♂ 2388 |  |  |  |  |  | (-2LOD) |  | (-2LOD) |  | (LOD max)^g^ |  |  |  |  |  | (LOD)^g^ |  |  |  |
| I |  | 2007 |  |  |  |  | 77111 |  |  | Outdoor (VES) |  | PC1 |  | 3.91-20.80 (0.0-26.59) |  | (6122g, 7554a, 9016a), **10001k; 6176b; 4550b, 1637e,** (7070a, 1870b, 3852n) |  | 5.4 (3.67) |  | 15.8 |  | 1637e |  | 5.4 (3.67) |  | 3.15 | 5.95 |
|  |  | 2007 |  |  |  |  | 77111 |  |  | Outdoor (VES) |  | PC3 |  | 9.33-20.80 (0.91-31.27) |  | (9016a), **10001k; 6176b; 4550b, 1637e,** (7070a, 1870b, 3852n, 10494v1) |  | 6 (3.76) |  | 15.8 |  | 1637e |  | 5.5 (3.72) |  | 3.25 | 4.35 |
|  |  | 2007 |  |  |  |  |  | 2388 |  | Outdoor (VES) |  | PC1 |  | 0-9.5 (0-15.69) |  | **10001k; 1637e, sb58e,** (7070e) |  | 6.3 (3.67) |  | 5.78 |  | 1637e |  | 5.2 (3.5) |  | 3.03 | 5.5 |
|  |  | 2007 |  |  |  |  |  | 2388 |  | Outdoor (VES) |  | PC3 |  | 0-14.37 (0-27.10) |  | **10001k; 1637e, sb58; 7070e,** (3852n, 10494e) |  | 5.6 (3.41) |  | 5.78 |  | 1637e |  | 5.1 (3.38) |  | 3.03 | 5.74 |
|  |  |  |  |  |  |  |  |  |  |  |  |  |  |  |  |  |  |  |  |  |  |  |  |  |  |  |  |
| II |  | 2006 |  |  |  |  | 77111 |  |  | Indoor (AAFC) |  | PC1 |  | 0.0-8.87 (0.0-14.76) |  | **14432m, 7530a,** (11151b, 11006a, 8177v1, 7492a) |  | 4.2 (4.11) |  | 3.87 |  | 7530a |  | 4.2 (4.03) |  | 3.18 | 4.17 |
|  |  | 2006 |  |  |  |  |  | 2388 |  | Indoor (AAFC) |  | PC1 |  | 0-10.0 (0-18.040) |  | **8781a,** (14432e; 8177v1) |  | 5 (4.33) |  | 0 |  | **8781a** |  | 5 (4.33) |  | 3.05 | 4.31 |
|  |  |  |  |  |  |  |  |  |  |  |  |  |  |  |  |  |  |  |  |  |  |  |  |  |  |  |  |
| III |  | 2005 |  | 80112 |  |  |  |  |  | Indoor (AAFC) |  | PC1 |  | 50.06-64.77 (50.06-64.77) |  | **Contig-3647-131, 9327e, 3870, 9889, 9863** |  | 10 (3.06) |  | 59.44 |  | 9863 |  | 8.4 (2.92 ns) |  | 3.02 | 4.51 |
|  |  | 2005 |  |  | 80109 |  |  |  |  | Indoor (AAFC) |  | PC1 |  | 32.59-51.18 (23.28-58.60) |  | (2473e, 1546m); **8456g;** **9327e; 3870; 9889; 9863** |  | 11.3 (3.41) |  | 36.7 |  | 1AFLP |  | 11.3 (3.41) |  | 3.07 | 4.82 |
|  |  | 2006 |  |  |  |  | 77111 |  |  | Indoor (AAFC) |  | PC1 |  | 77.02-115.48 (71.02-152.41) |  | **12531a, 10460a; sb62b; 7398b; 9027f; 6517j; 5488j; 3713a, 10659-2, 2239a, 10660a, 13661a, 9147b, 2473e, 10759k** (8456j, 14683a, 9889e, 9863a, 14745e, 5952m) |  | 3.9 (3.89) |  | 105.83 |  | 10660a |  | 3.9 (3.89) |  | 2.96 | 4.17 |
|  |  | 2006 |  |  |  |  | 77111 |  |  | Outdoor (VES) |  | PC1 |  | 104.56-106.54 107.38-114.48 (82.02-116.48) |  | (12531a, 10460a; sb62b; 7398b; 9027f; 6517j; 5488j; 3713a, 10659-2, 2239a), **10660a,** (13661a, 9147b), **2473e, 10759k** |  | 3.9 (4.03) |  | 110.48 |  | 10759k |  | 3.3-3.9 (3.35-4.03) |  | 2.94 | 4.7 |
|  |  | 2006 |  |  |  |  |  | 2388 |  | Indoor (AAFC) |  | PC1 |  | 55.56-80.78 (52.10-86.14) |  | (12531e), **5488j, 9027f, 6517j, sb62f, 10460e, 10129, 7398f, 3713d, 10659-2, 2239a; 10660e; 9147b; 2473f; 15280e; 10759e;** (1546e, 8456j) |  | 3.6 (3.73) |  | 73.39 |  | 9147b |  | 3.6 (3.73) |  | 2.95 | 4.31 |
|  |  | 2007 |  |  |  |  |  | 2388 |  | Indoor (AAFC) |  | PC1 |  | 95.68-129.24 (89.68-129.24) |  | **9889b, 3870f, 9327f, 9863e, 10981a, 3393f, 6971j, 14473f, 10240e, 9629e,5953g, 2002e** |  | 6.6 (3.12) |  | 100.72 |  | 9863e |  | 6.6 (3.11) |  | 3 | 4.7 |
|  |  | 2006 |  |  |  |  |  | 2388 |  | Outdoor (VES) |  | PC1 |  | 69.64-79.78 (50.10-87.14) |  | (12531e, 5488j), **9027f, 6517j, sb62f, 10460e, 10129, 7398f, 3713d**, (10659-2, 2239a); **10660e; 9147b; 2473f; 15280e; 10759e;** (1546e, 8456j) |  | 3.3 (3.26) |  | 75.78 |  | 10759e |  | 3.3 (3.26) |  | 2.85 | 4.9 |
|  |  |  |  |  |  |  |  |  |  |  |  |  |  |  |  |  |  |  |  |  |  |  |  |  |  |  |  |
| IV |  | 2005 |  |  | 80109 |  |  |  |  | Indoor (AAFC) |  | PC1 |  | 101.97-122.89 (96.01-122.89) |  | **9593; 14337, Sb08** |  | 12.7 (3.63) |  | 121.28 |  | sb08 |  | 10.9 (3.36) |  | 2.95 | 4.82 |
|  |  | 2005 |  | 80112 |  |  |  |  |  | Indoor (AAFC) |  | PC1 |  | 48.19-52.76 (43.19-65.48) |  | **b123, 9593** (14337) |  | 10.7 (3.66) |  | 48.88 |  | b123 |  | 10.7 (3.66) |  | 3.01 | 4.51 |
|  |  |  |  |  |  |  |  |  |  |  |  |  |  |  |  |  |  |  |  |  |  |  |  |  |  |  |  |
| V |  | 2006 |  |  |  |  | 77111 |  |  | Indoor (AAFC) |  | PC1 |  | 59.21-84.62 (46.89-169.95) |  | (11390a);  **7050a, 14023a,** (15115e, 15170h,b91c, 14088a, 8398a, 6647a, 9630m, 10797-4, 6718e, 6922b), ***sb21a, 3720j***, (10732v1, 0470a, 9644v2; 7242a, 6580a, 10394t2; 14320b; 9406b, 12722j; 10140e; 9898a, 2343e, 13749d, 7248e, 7937b) |  | 6 (4.75) |  | 63.95 |  | **7050a** |  | 6.1(4.5) |  | 2.8 | 4.17 |
|  |  | 2006 |  |  |  |  |  | 2388 |  | Indoor (AAFC) |  | PC1 |  | 78.21-94.19 (44.50-135.52) |  | (Sb16mh, 2654e, 0309g, b91e, 14088m, 15170g, 8398m, 6647e, 6718e), **6922a, 7178a, 10049e,** (Sb21e, 3720j, 10732v1, 9644v2); ***12255f, 10394t2; 5873t1; 14320e***; (10140e; 12722i; 2343j, 13749k, 7248f) |  | 4.5 (3.61) |  | 81.73 |  | 10049e |  | 3.5 (3.22) |  | 2.77 | 4.31 |
|  |  | 2006 |  |  |  |  | 77111 |  |  | Indoor (AAFC) |  | PC1 |  | 102.68-126.22 (46.89-169.95) |  | (11390a);  ***7050a, 14023a,*** (15115e, 15170h,b91c, 14088a, 8398a, 6647a, 9630m, 10797-4, 6718e, 6922b), **sb21a, 3720j**, (10732v1, 0470a, 9644v2; 7242a, 6580a, 10394t2; 14320b; 9406b, 12722j; 10140e; 9898a, 2343e, 13749d, 7248e, 7937b) |  | 6 (4.75) |  | 121.47 |  | sb21a |  | 3.1 (3.09) |  | 2.8 | 4.17 |
|  |  | 2006 |  |  |  |  |  | 2388 |  | Indoor (AAFC) |  | PC1 |  | 113.37-130.04 (44.50-135.52) |  | (Sb16mh, 2654e, 0309g, b91e, 14088m, 15170g, 8398m, 6647e, 6718e), ***6922a, 7178a, 10049e,*** (Sb21e, 3720j, 10732v1, 9644v2); **12255f, 10394t2; 5873t1; 14320e**; (10140e; 12722i; 2343j, 13749k, 7248f) |  | 4.5 (3.61) |  | 116.38 |  | 12255f |  | 3.3 (3.41) |  | 2.77 | 4.31 |
|  |  |  |  |  |  |  |  |  |  |  |  |  |  |  |  |  |  |  |  |  |  |  |  |  |  |  |  |
| VI |  | 2005 |  | 80112 |  |  |  |  |  | Indoor (AAFC) |  | PC1 |  | 12.88-26.71 (3.91-26.71) |  | (6798, 8374), **4220, 7245** |  | 14.2 (3.75) |  | 18.90-20.28 |  | 4220-7245 |  | 9.2 (3.27) |  | 3.27 | 4.51 |
|  |  | 2005 |  | 80112 |  |  |  |  |  | Indoor (AAFC) |  | PC2 |  | 56.60-69.97 (40.34-75.97) |  | **90132** |  | 9.5 (3.38) |  | 66.2 |  | 90132 |  | 9.5 (3.38) |  | 3.25 | 4.47 |
|  |  | 2004 |  | 80112 |  |  |  |  |  | Outdoor (VES) |  | PC1 |  | 34.96-39.83 (22.28-48.34) |  | (Contig-1801-232, 8902b), **1687** |  | 6.1 (3.04 ns) |  | 36.83 |  | 1687 |  | 6.1 (3.04 ns) |  | 3.3 | 5.72 |
|  |  | 2005 |  |  | 80109 |  |  |  |  | Indoor (AAFC) |  | PC1 |  | 8.18-38.13 (2.0-55.32) |  | (M017, 90008f), **6798v1, 14017e, 8374, 4220, 7245, 10034v1,** (1687, Contig-3186-280, 10583v1, 7604) |  | 9.1 (3.23) |  | 25.28 |  | 7245 |  | 9.1 (3.23) |  | 3.12 | 4.82 |
|  |  | 2005 |  |  | 80109 |  |  |  |  | Indoor (AAFC) |  | PC2 |  | 58.32-77.15 (6.18-81.15) |  | (6798v1, 14017e, 8374, 4220, 7245, 10034v1, 1687, Contig-3186-280, 10583v1, 7604); **90132** |  | 9.4 (3.35) |  | 71.27 |  | 90132 |  | 9.4 (3.35) |  | 3.15 | 4.55 |
|  |  | 2004 |  |  | 80109 |  |  |  |  | Outdoor (VES) |  | PC2 |  | 33.28-43.42 (29.28-85.15) |  | **10034v1, 1687, Contig-3186-280,** (10583v1, 7604, 90132) |  | 7.7 (3.41) |  | 37.12 |  | 10034v1 |  | 7.4 (3.23 ns) |  | 3.26 | 4.45 |
|  |  | 2006 |  |  |  |  | 77111 |  |  | Indoor (AAFC) |  | PC1 |  | 115.26-140.03 (105.42-146.63) |  | (4205a, 5463f, 9870t3, 7864k); **2940a,** (7115c) |  | 6.8 (4.67) |  | 118.03 |  | 2940a |  | 4.1 (4.06) |  | 2.75 | 4.17 |
|  |  | 2006 |  |  |  |  | 77111 |  |  | Outdoor (VES) |  | PC1 |  | 74.93-131.03 (74.17-144.03) |  | (7106p1), **8083a, 10016v1, 10328a, 5345a, 7183a, 12414j, 14898a; 14528a; 5325j; 2980b, 7919j; 5435b, 4205a, 5463f, 9870t3, 7864k, 2940a** |  | 2.9 (2.72 ns) |  | 90.31 |  | 7183a |  | 2.9 (2.69 ns) |  | 2.75 | 4.7 |
|  |  | 2007 |  |  |  |  | 77111 |  |  | Outdoor (VES) |  | PC1 |  | 95.89-109.73 (90.31-138.03) |  | (12414j), **14898a; 14528a; 5325j; 2980b, 7919j; 5435b, 4205a**, (5463f, 9870t3, 7864k, 2940a) |  | 4.3 (2.91) |  | 102.30-102.42 |  | 7919j; 5435b |  | 4.3 (2.91) |  | 2.77 | 5.95 |
|  |  | 2007 |  |  |  |  | 77111 |  |  | Outdoor (VES) |  | PC1 |  | 93.89-108.73 (85.21-144.63) |  | (5345a, 7183a, 12414j), **14898a; 14528a; 5325j; 2980b, 7919j; 5435b, 4205a**, (5463f), **9870t3, 7864k, 2940a** |  | 4.9 (3.17) |  | 97.41 |  | 14898a |  | 4.8 (3.16) |  | 3.06 | 4.32 |
|  |  | 2006 |  |  |  |  |  | 2388 |  | Indoor (AAFC) |  | PC1 |  | 80.73-110.26 (71.99-125.96) |  | (5325j, 7919j, 5435e, 2980e, 10161e); **10211f; 5463j, sb60f; 11166e; 7864n; 9870t4, 3199e, sb24h, 7115f** |  | 3.4 (3.48) |  | 83.91 |  | 5463i |  | 3.4 (3.48) |  | 3 | 4.31 |
|  |  | 2006 |  |  |  |  |  | 2388 |  | Outdoor (VES) |  | PC1 |  | 68.45-73.99 (56.59-93.99) |  | (10016j, 5345a, 12414e), **2058e, 7343e, 14898f, 14528e,** (5325j, 7919j, 5435e; 2980e; 10161e**;** 10211f; 5463j, sb60f, 11166e, 7864n, 9870t4, 3199e) |  | 3.6 (3.57 ns) |  | 70.18 |  | 7343e |  | 3.6 (3.57 ns) |  | 3.76 | 4.9 |
|  |  | 2007 |  |  |  |  |  | 2388 |  | Outdoor (VES) |  | PC1 |  | 74.99-83.05 (70.18-125.96) |  | (14898f, 14528e), **5325j; 7919j, 5435e**; **2980e; 10161e;** (10211f; 5463j, sb60f, 11166e, 7864n, 9870t4, 3199e, sb24h, 7115f) |  | 4.7 (3.11) |  | 79.29 |  | 5435e |  | 4.7 (3.11) |  | 3.06 | 4.39 |
|  |  |  |  |  |  |  |  |  |  |  |  |  |  |  |  |  |  |  |  |  |  |  |  |  |  |  |  |
| VIII |  | 2005 |  | 80112 |  |  |  |  |  | Indoor (AAFC) |  | PC1 |  | 50.54-57.77 (37.51-57.77) |  | (15051a, 10125), **7053v2, PAAC19** |  | 13.1 (3.83) |  | 51.33 |  | 7053v2 |  | 13.1 (3.83) |  | 3.27 | 4.51 |
|  |  | 2005 |  |  | 80109 |  |  |  |  | Indoor (AAFC) |  | PC1 |  | 41.54-50.98 (39.54-58.17) |  | **PAAC19** (Contig-1318-285) |  | 11.6 (3.83) |  | 46.11 |  | PAAC19 |  | 10.7 (3.82) |  | 3.14 | 4.82 |
|  |  | 2004 |  |  | 80109 |  |  |  |  | Outdoor (VES) |  | PC2 |  | 22.80-33.38 (13.83-35.09) |  | (15609e), **7977f** |  | 8.7 (3.35) |  | 29.57; 31.38 |  | 1AFLP; 7977f |  | 8.7; 7.5 (3.35; 2.88) |  | 3.09 | 4.45 |
|  |  | 2007 |  |  |  |  | 77111 |  |  | Outdoor (VES) |  | PC2 |  | 14-50-19.97; 25.61-59.51 (3.72-88.61) |  | (1810a, 0835a, 15609b, 7548a, 7977a, 5102a, 6479k), **9829,** (15051j), **10125n; 2909d, 1084a; 10409b; 3773c; 0167e,** (14723b, 9006a, 2380a, 12402g, 8406c, 5994a) |  | 5.1 (3.21) |  | 16.97; 33.67 |  | 9829; 2909d |  | 4.5; 5.1 (3.07; 3.21) |  | 3.07 | 4.25 |
|  |  | 2007 |  |  |  |  |  | 2388 |  | Outdoor (VES) |  | PC2 |  | 16.44-20.61; 23.33-52.90 (8.96-63.37) |  | (6479k, 7977f); **9829, 7964a**; (15051j, 0186f); **10125n, 5068f; 10409e; 0167e; 6490m; 1289g; 14723e**, (12402g) |  | 6.5 (3.36) |  | 28.2 |  | 10125n |  | 4.5; 4.8 (3.26) |  | 3.17 | 4.29 |
|  |  | 2007 |  |  |  |  |  | 2388 |  | Outdoor (VES) |  | PC3 |  | 30.48-52.90 (22.33-63.37) |  | (10125n, 5068f), **10409e, 0167e, 6490m, 1289g,** (14723e, 12402g) |  | 6 (3.14) |  | 45.59 |  | 0167e |  | 4 (2.68 ns) |  | 3.06 | 5.74 |
|  |  |  |  |  |  |  |  |  |  |  |  |  |  |  |  |  |  |  |  |  |  |  |  |  |  |  |  |
| X |  | 2006 |  |  |  |  |  | 2388 |  | Indoor (AAFC) |  | PC1 |  | 0.0-4.51 (0.0-23.78) |  | **4914j, 4341e-2, 12821a; 4794f, 6053e; 5440e;** (6903p1; 10698f, 8806a, 90003m, 10355v3, 90019f) |  | 2.7 (2.8) |  | 0.96 |  | 4794f |  | 2.7 (2.8) |  | 2.69 | 4.31 |
|  |  | 2006 |  |  |  |  |  | 2388 |  | Outdoor (VES) |  | PC1 |  | 2.96-18.78 (0.0-45.92) |  | (4914j, 4341e-2, 12821a, 4794f), **6053e, 5440e, 6903p1, 10698f, 8806a, 90003m,** (10355v3, 90019f, 4543e, 5957t1, 6593e, 9121m, 6349e) |  | 3.9 (3.54) |  | 4.81 |  | 6903p1 |  | 3.9 (3.54) |  | 2.68 | 4.9 |
|  |  | 2005 |  | 80112 |  |  |  |  |  | Indoor (AAFC) |  | PC1 |  | 120.75-122.96 (120.35-123.35) |  | **b5** |  | 22.2 (8.13) |  | 121.96 |  | **b5** |  | 20.9 (7.7) |  | 2.92 | 4.51 |
|  |  | 2006 |  |  |  |  | 77111 |  |  | Indoor (AAFC) |  | PC1 |  | 84.24-130.97 (84.24-130.97) |  | **7914a; 7736b, 5437a, 1686c, 2871a; 8228m, 4148e; 5731j** |  | 6.8 (2.87) |  | 107.06 |  | 5437a |  | 6.8 (2.87) |  | 2.41 | 4.17 |
|  |  | 2006 |  |  |  |  |  | 2388 |  | Indoor (AAFC) |  | PC1 |  | 87.54-111.36 (70.72-111.36) |  | (7914h, 7235e, 7736e, 10998e, 4348f), **10799e;8228f;3163e, 13950e** |  | 2.5 (2.64 ns) |  | 111.36 |  | 13950e |  | 2.5 (2.64 ns) |  | 2.69 | 4.31 |
|  |  |  |  |  |  |  |  |  |  |  |  |  |  |  |  |  |  |  |  |  |  |  |  |  |  |  |  |
| XI |  | 2006 |  |  |  |  | 77111 |  |  | Outdoor (VES) |  | PC1 |  | 88.90-116.99 (75.75-120.99) |  | (7073k,), **14765a, 4415a; 7465e, 10545a, 5090v1; 0008b, 8001k; 16279c; 9573t1,** |  | 3.4 (3.41) |  | 98.42 |  | 0008b |  | 3.3 (3.39) |  | 3.13 | 4.7 |
|  |  | 2006 |  |  |  |  |  | 2388 |  | Outdoor (VES) |  | PC1 |  | 88.91-113.39 (80.08-119.39) |  | (7073j, 14862m, 4415j); **8987p1; 5090v1, 8001k; 4711f; 9573t1**, (7112e) |  | 3.5 (3.38) |  | 94.86 |  | 5090v1 |  | 3.2 (3.27) |  | 3.02 | 4.9 |
|  |  |  |  |  |  |  |  |  |  |  |  |  |  |  |  |  |  |  |  |  |  |  |  |  |  |  |  |
| XII |  | 2006 |  |  |  |  | 77111 |  |  | Indoor (AAFC) |  | PC1 |  | 65.163-93.245 (54.850-98.245) |  | (8656p1, 1471m), **4705j, 10965b, 3706j, 15209a; 2260a, 12347a** |  | 3.1 (3.21) |  | 85.64 |  | 2260a |  | 3.1 (3.21) |  | 2.95 | 4.17 |
|  |  | 2007 |  |  |  |  | 77111 |  |  | Indoor (AAFC) |  | PC1 |  | 97.25-105.42 (94.25-106.32) |  | **6569a, 15328f,** (9240) |  | 13.3 (8.38) |  | 103.66 |  | **15328f** |  | 13.3 (8.38) |  | 2.8 | 4.15 |
|  |  | 2007 |  |  |  |  | 77111 |  |  | Outdoor (VES) |  | PC2 |  | 94.25-107.75; 108.72-121.64 (88.25-130.53) |  | **6569a, 15328f, 9240, 9065c, sb64a,** (15817j, 4113a, 0901a),OK, (9163t1, 3656v1, 9955j) |  | 10.9 (6.17) |  | 103.66 |  | **15328f** |  | 8.2 (5.65) |  | 2.98 | 4.25 |
|  |  | 2007 |  |  |  |  | 77111 |  |  | Outdoor (VES) |  | PC3 |  | 110.72-128.08 (71.76-132.29) |  | (3706j, 15209a, 2260a), **12347a, 6569a,** (15328f, 9240, 9065c, sb64a, 15817j, 4113a, 0901a), **9163t1, 3656v1** (9955j, 5535n, b141a, 4995c, 6523a, 12214b) |  | 7 (3.68) |  | 121.64 |  | 9163t1 |  | 5 (3.34) |  | 2.9 | 4.35 |
|  |  | 2007 |  |  |  |  |  | 2388 |  | Indoor (AAFC) |  | PC1 |  | 94.04-102.83 (91.04-113.64) |  | **6677m, 15328f, Sb64e,** (15817j, 9665f) |  | 14.4 (8.35) |  | 98.71 |  | **6677m** |  | 13 (8.12) |  | 2.94 | 4.7 |
|  |  | 2007 |  |  |  |  |  | 2388 |  | Outdoor (VES) |  | PC2 |  | 103.83-114.64 (83.04-122.18) |  | (6677m, 15328f, sb64e, 15817j), **9665f,** (3656v1, 10703e,9955a) |  | 11.7 (6.67) |  | 107.64 |  | **9665f** |  | 11.5 (6.6) |  | 2.87 | 4.29 |
|  |  | 2007 |  |  |  |  |  | 2388 |  | Outdoor (VES) |  | PC3 |  | 104.83-118.48 (102.83-122.19) |  | **9665f, 3656v1; 10703e,** (9955a, 5535e) |  | 8.1 (4.18) |  | 107.64 |  | 9665f |  | 7.8 (4.03) |  | 3 | 5.74 |

^a^ Two different colors of green distinguish the individual QTLs associated at two QTL-regions identified onto the same linkage group.

^b^ Years of measurement: 2004 and 2005 for the mapping population *P* and, 2006 and 2007 for the mapping population *D*.

^c^ Environmental conditions tested: in a large growth chamber (i.e. indoor conditions) at Agriculture and Agri-Food Canada (AAFC) under a declining photoperiod and day/night-time temperatures of 24/15°C; at Valcartier Experimental Station (VES) under natural outdoor conditions.

^d^ Principal component retained after PCA (see § Materials and Methods; Table 2).

^e^ Confidence interval of each QTL calculated by one-LOD and two-LOD below the QTL-LOD peak (i.e. at -1LOD and -2LOD).

^f^ Proportion of phenotypic variance explained.

^g^ ns = non significant.
