# Supplementary material for: QTL mapping in white spruce: gene maps and genomic regions underlying adaptive traits across pedigrees, years and environments
Source: BMC Genomics. 2011 Mar 10;12:145. doi: 10.1186/1471-2164-12-145 (PMC3068112; doi:10.1186/1471-2164-12-145)
Supplement: Additional file 6 — QTLs identified for height growth. List of QTLs identified for growth within each mapping population, P and D, for each environmental condition and year (QTL interval position, PPVE percent of phenotypic variance explained, LOD value, list of gene loci for each QTL). [file 1471-2164-12-145-S6.DOC]

**Additional file 6**. QTLs identified for height growth.

|  |  |  |  | Mapping populations | | | | |  | Environmental conditions^c^ |  | Measure^d^ |  | QTL interval^e^ | | | | | | |  | | Peak gene | | | | | | |  | LOD threshold | |
| --- | --- | --- | --- | --- | --- | --- | --- | --- | --- | --- | --- | --- | --- | --- | --- | --- | --- | --- | --- | --- | --- | --- | --- | --- | --- | --- | --- | --- | --- | --- | --- | --- |
| Linkage group^a^ |  | Year^b^ |  | *P*  (C96-1-2856) | |  | *D*  (C94-1-2516) | |  |  |  |  |  | Position at -1LOD |  | Gene loci at -1LOD |  | | PPVE^f^ | |  | | Position in cM | |  | | Marker-SNP |  | PPVE^f^ |  | At linkage group level | At genome level |
|  |  |  |  | ♀ 80112 | ♂ 80109 |  | ♀ 77111 | ♂ 2388 |  |  |  |  |  | (-2LOD) |  | (-2LOD) |  | | (LOD max)^g^ | |  | |  | |  | |  |  | (LOD)^g^ |  |  |  |
| **I** |  | 2005 |  | 80112 |  |  |  |  |  | Indoor (AAFC) |  | A |  | 189.80-198.47 (185.80-198.47) |  | **6250** |  | 10.5 (3.57) | |  | | 198.47 | |  | | 6250 | |  | 10.1 (3.56) |  | 3.1 | 4.41 |
|  |  | 2005 |  |  | 80109 |  |  |  |  | Indoor (AAFC) |  | A |  | 192.03-207.80 (186.02-207.80) |  | (Contig-2118-393), **3579, 6250, 7561e** |  | | 10 (3.5) | |  | | 199.16 | |  | | 7561e |  | 10 (3.5) |  | 3.01 | 4.56 |
|  |  | 2007 |  |  |  |  | 77111 |  |  | Outdoor (VES) |  | A |  | 131.57-165.8 (114.76-171.4) |  | (10727a), **16364a, 2114a, 12694g,** (7488a) |  | | 5.1 (3.96) | |  | | 149.21 | |  | | 16364a |  | 4.9 (3.94) |  | 3.18 | 4.22 |
|  |  | 2005 |  |  |  |  |  | 2388 |  | Indoor (AAFC) |  | A |  | 112.29-135.60 (100.29-152.03) |  | (10558-1), **16364f, 2114e, 11066j,** (12694g, 3579e, 6250e, 817e) |  | | 3.4 (2.98 ns) | |  | | 131.01 | |  | | 2114e |  | 2.7 (2.72 ns) |  | 3.02 | 4.25 |
|  |  | 2006 |  |  |  |  |  | 2388 |  | OUTDOOR (VES) |  | T |  | 125.46-142.87 (116.50-152.56) |  | (16364f), **2114e, 11066j, 12694g,** (3579e, 6250e, 817e, 7488e) |  | | 3.6 (3.31) | |  | | 131.01 | |  | | 2114e |  | 3.6 (3.31) |  | 3.04 | 4.29 |
|  |  | 2007 |  |  |  |  |  | 2388 |  | OUTDOOR (VES) |  | A |  | 131.60-152.56 (91.59-152.56) |  | (1378b, 11089e, 10558-1, 16364f, 2114e, 11066j), **12694g, 3579e, 6250e, 817e, 7488e** |  | | 3.5 (3.01) | |  | | 147.03 | |  | | 817e |  | 3.5 (3.01) |  | 2.99 | 4.2 |
|  |  |  |  |  |  |  |  |  |  |  |  |  |  |  |  |  |  | |  | |  | |  | |  | |  |  |  |  |  |  |
| **II** |  | 2007 |  |  |  |  | 77111 |  |  | Indoor (AAFC) |  | A |  | 138.94-145.94 (135.87 -173.91) |  | (16228a), **2779a, 10642-1, 12575b,** (5280j, 7393a, 4141a, 6723a, 2202e, 5321m) |  | | 4.7 (5.14) | |  | | 143.82 | |  | | **10642-1** |  | 4.7 (5.14) |  | 2.97 | 4.2 |
|  |  | 2007 |  |  |  |  |  | 2388 |  | Indoor (AAFC) |  | A |  | 121.49-128.09 (120.49-144.30) |  | **00012e, 10642-1, 9061e,** (5280m, 7393a, 3376v1, 6723e) |  | | 5.1 (5.22) | |  | | 125.33 | |  | | **00012e** |  | 5.1 (5.22) |  | 3.19 | 4.29 |
|  |  |  |  |  |  |  |  |  |  |  |  |  |  |  |  |  |  | |  | |  | |  | |  | |  |  |  |  |  |  |
| **III** |  | 2005 |  |  |  |  | 77111 |  |  | INDOOR (AAFC) |  | A |  | 95.68-105.56 (90.80-133.29) |  | (3713a), **10659-2, 2239a,** (10660a, 13661a, 9147b, 2473e, 10759k, 8456j, 14683a) |  | | 6.4 (6.89) | |  | | 101.29 | |  | | **10659-2** |  | 6.4 (6.89) |  | 2.79 | 4.07 |
|  |  | 2006 |  |  |  |  | 77111 |  |  | INDOOR (AAFC) |  | T |  | 110.48-124.81 (99.68-133.29) |  | (10659-2, 2239a,10660a, 13661a, 9147b, 2473e, 10759k), **8456j,** (14683a) |  | | 7.7 (7.73) | |  | | 117.81 | |  | | **8456j** |  | 7.2 (7.61) |  | 3.17 | 4.2 |
|  |  | 2007 |  |  |  |  | 77111 |  |  | INDOOR (AAFC) |  | A |  | 103.56-110.48 (95.68-144.31) |  | (10659-2, 2239a), **10660a, 13661a, 9147b, 2473e,** (10759k, 8456j, 14683a, 9889e, 9863a, 14745e) |  | | 5.1 (5.53) | |  | | 109.64 | |  | | **2473e** |  | 5.1 (5.53) |  | 3.02 | 4.2 |
|  |  | 2005 |  |  |  |  | 77111 |  |  | OUTDOOR (VES) |  | A |  | 110.48-123.81 (92.80-127.29) |  | (3713a, 10659-2, 2239a, 10660a, 13661a, 9147b, 2473e, 10759k) **8456j,** (14683a) |  | | 7.5 (7.79) | |  | | 117.81 | |  | | **8456j** |  | 7.5 (7.79) |  | 2.99 | 4.09 |
|  |  | 2006 |  |  |  |  | 77111 |  |  | OUTDOOR (VES) |  | T |  | 95.68-130.29 (69.02-144.31) |  | (12531a, 10460a, sb62b, 7398b, 9027f, 6517j, 5488j, 3713a), **10659-2, 2239a, 10660a, 13661a, 9147b, 2473e, 10759k, 8456j, 14683a**, (9889e, 9863a, 14745e) |  | | 4.3 (4.15) | |  | | 117.81 | |  | | 8456j |  | 4.1 (4.13) |  | 3.04 | 4.18 |
|  |  | 2005 |  |  |  |  |  | 2388 |  | INDOOR (AAFC) |  | A |  | 64.51-96.64 (52.56-96.64) |  | (5488j, 9027f, 6517j, Sb62f, 10460e, 10129, 7398f, 3713d), **10659-2, 2239a, 10660e, 9147b, 2473f, 15280e, 10759e, 1546e, 8456j, 14683e** |  | | 6.3 (6.48) | |  | | 88.68 | |  | | **14683e** |  | 7.8 (6.39) |  | 3.01 | 4.25 |
|  |  | 2006 |  |  |  |  |  | 2388 |  | INDOOR (AAFC) |  | T |  | 73.39-96.64 (67.51-96.64) |  | (10659-2, 2239a, 10660e, 9147b), **2473f, 15280e, 10759e, 1546e, 8456j, 14683e** |  | | 7.3 (7.42) | |  | | 80.97 | |  | | **1546e** |  | 7.1 (7.36) |  | 3 | 4.2 |
|  |  | 2007 |  |  |  |  |  | 2388 |  | INDOOR (AAFC) |  | A |  | 71.99-85.14 (66.51-97.36) |  | (10659-2, 2239a, 10660e), **9147b, 2473f, 15280e, 10759e, 1546e, 8456j,** (14683e, 9889b, 3870f) |  | | 5.6 (5.59) | |  | | 75.78 | |  | | **10759e** |  | 5.3 (5.46) |  | 2.99 | 4.29 |
|  |  | 2005 |  |  |  |  |  | 2388 |  | OUTDOOR (VES) |  | A |  | 71.99-88.14 (61.51-92.68) |  | (3713d, 10659-2, 2239a), **10660e, 9147b, 2473f, 15280e, 10759e, 1546e, 8456j,** (14683e) |  | | 7.1 (7.46) | |  | | 74.47 | |  | | **2473f** |  | 6.7 (7.38) |  | 3.04 | 4.29 |
|  |  | 2006 |  |  |  |  |  | 2388 |  | OUTDOOR (VES) |  | T |  | 74.39-88.68 (64.51-96.64) |  | (10659-2, 2239a, 10660e, 9147b), **2473f, 15280e, 10759e, 1546e, 8456j,** (14683e) |  | | 4.5 (4.8) | |  | | 80.97 | |  | | **1546e** |  | 4.5 (4.8) |  | 3.11 | 4.29 |
|  |  | 2007 |  |  |  |  |  | 2388 |  | OUTDOOR (VES) |  | A |  | 70.64-97.36 (63.51-103.72) |  | (3713d, 10659-2, 2239a), **10660e, 9147b, 2473f, 15280e, 10759e, 1546e, 8456j, 14683e, 9889b, 3870f,** (9327f, 9863e) |  | | 3.3 (3.22) | |  | | 83.14 | |  | | 8456j |  | 3 (3.2) |  | 3 | 4.2 |
|  |  |  |  |  |  |  |  |  |  |  |  |  |  |  |  |  |  | |  | |  | |  | |  | |  |  |  |  |  |  |
| **IV** |  | 2006 |  |  |  |  | 77111 |  |  | Indoor (AAFC) |  | T |  | 10.41-26.70 (5.65-37.64) |  | (4613c), **650b, 8984c,** (sb32d, 10429) |  | | 8.2 (3.41) | |  | | 16.7 | |  | | 8984c |  | 6.8 (3.18) |  | 2.96 | 4.2 |
|  |  | 2005 |  |  |  |  |  | 2388 |  | Indoor (AAFC) |  | A |  | 9.45-46.51 (0.0-55.06) |  | (b54k, 10193e, 14265a, 7005e, b140m, 10438f, 9693v3, 4045h, 4613k), **650e, 1529e** |  | | 6.6 (3.04) | |  | | 11.01 | |  | | 0650e |  | 2.4 (2.21 ns) |  | 2.8 | 4.25 |
|  |  | 2005 |  |  |  |  | 77111 |  |  | OUTDOOR (VES) |  | A |  | 72.45-104.71 (47.46-151.78) |  | (9780a, 12871a, 10378v1, 4632v1, 10060c2, 3673a, 16369a), **5720j, 4700f, 5106a, b123a, 1312a,** (14337j, 2931j, sb08b, 9514a, 7857t1, 4962a, 4562c) |  | | 3 (2.89) | |  | | 87.31 | |  | | 5720j |  | 2.6 (2.86) |  | 2.88 | 4.09 |
|  |  | 2005 |  |  |  |  |  | 2388 |  | OUTDOOR (VES) |  | A |  | 68.53-101.75 (57.30-106.91) |  | (16369b), **5720j, 4700f, 10254g, 4642g, 9593e, 3491f, 1312a, 701e, 10414e,** (14337e, 2931j, 10554j) |  | | 3 (2.93) | |  | | 86.2 | |  | | 4700f, 5720j |  | 2.6 (2.9) |  | 2.86 | 4.29 |
|  |  |  |  |  |  |  |  |  |  |  |  |  |  |  |  |  |  | |  | |  | |  | |  | |  |  |  |  |  |  |
| **V** |  | 2006 |  |  |  |  | 77111 |  |  | Indoor (AAFC) |  | A |  | 66.95-97.45 (59.21-107.68) |  | (7050a), **14023a, 15115e, 15170h, b91c, 14088a, 8398a, 6647a, 9630m,** (10797-4, 6718e, 6922b) |  | | 3.8 (4.02) | |  | | 93.13 | |  | | 8398a |  | 3.8 (4.02) |  | 2.9 | 4.18 |
|  |  | 2006 |  |  |  |  | 77111 |  |  | Indoor (AAFC) |  | T |  | 60.21-103.68 (31.69-114.68) |  | (6342a, 2510b, 11390a), **7050a, 14023a, 15115e, 15170h, b91c, 14088a, 8398a, 6647a, 9630m,** **10797-4, 6718e, 6922b** |  | | 3.5 (3.63) | |  | | 93.39 | |  | | 8398a |  | 3.5 (3.63) |  | 2.77 | 4.2 |
|  |  | 2007 |  |  |  |  | 77111 |  |  | Indoor (AAFC) |  | A |  | 39.89-83.62 (31.69-95.39) |  | (6342a), **2510b, 11390a, 7050a, 14023a,** (15115e, 15170h, b91c, 14088a, 8398a, 6647a) |  | | 7.1 (4.64) | |  | | 71.62 | |  | | **14023a** |  | 7.1 (4.64) |  | 2.87 | 4.2 |
|  |  | 2006 |  |  |  |  | 77111 |  |  | OUTDOOR (VES) |  | A |  | 64.95-81.62 (56.52-88.62) |  | (11390a, 7050a), **14023a** |  | | 2.9 (3.14) | |  | | 71.62 | |  | | 14023a |  | 2.9 (3.14) |  | 2.89 | 4.25 |
|  |  | 2007 |  |  |  |  | 77111 |  |  | OUTDOOR (VES) |  | A |  | 33.89-75.62 (31.69-79.62) |  | (6342a), **2510b, 11390a, 7050a, 14023a** |  | | 6.2 (4.64) | |  | | 71.62 | |  | | **14023a** |  | 5.1 (4.42) |  | 2.93 | 4.22 |
|  |  | 2006 |  |  |  |  |  | 2388 |  | Indoor (AAFC) |  | A |  | 41.86-76.24 (17.53-81.73) |  | (7114e), **2510f, sb16Mh, 2654e, 309g, b91e, 14088m, 15170g, 8398m, 6647e,** (6718e, 6922a, 7178a) |  | | 4.1 (4.09) | |  | | 67.17 | |  | | 0309g |  | 4.1 (4.09) |  | 2.85 | 4.25 |
|  |  | 2006 |  |  |  |  |  | 2388 |  | Indoor (AAFC) |  | T |  | 54.05-83.73 (17.53-90.73) |  | (7114e, 2510f, sb16Mh, 2654e), **309g, b91e, 14088m, 15170g, 8398m, 6647e, 6718e, 6922a, 7178a, 10049e** |  | | 3 (3.19) | |  | | 70.74 | |  | | 15170 |  | 3 (3.19) |  | 2.87 | 4.2 |
|  |  | 2007 |  |  |  |  |  | 2388 |  | Indoor (AAFC) |  | A |  | 41.86-45.28 (25.86-73.24) |  | **2510f,** (sb16Mh, 2654e, 309g, b91e, 14088m, 15170g, 8398m, 6647e) |  | | 9.2 (4.47) | |  | | 42.498 | |  | | 2510f |  | 9.2 (4.47) |  | 2.82 | 4.29 |
|  |  | 2005 |  |  |  |  | 77111 |  |  | INDOOR (AAFC) |  | A |  | 154.71-169.95 (148.71-169.95) |  | **9406b, 12722j, 10140e, 9898a, 2343e, 13749d, 7248e, 7937b** |  | | 4.5 (4.45) | |  | | 165.35 | |  | | **13749d** |  | 4.4 (4.42) |  | 2.74 | 4.07 |
|  |  | 2006 |  |  |  |  | 77111 |  |  | INDOOR (AAFC) |  | T |  | 151.71-169.95 (142.09-169.95) |  | (6580a, 10394t2, 14320b), **9406b, 12722j, 10140e, 9898a, 2343e, 13749d, 7248e, 7937b** |  | | 4.5 (4.46) | |  | | 160.35 | |  | | **2343e** |  | 4.3 (4.45) |  | 2.77 | 4.2 |
|  |  | 2005 |  |  |  |  | 77111 |  |  | OUTDOOR (VES) |  | A |  | 152.71-167.35 (147.71-169.95) |  | **9406b, 12722j, 10140e, 9898a, 2343e, 13749d**, (7248e, 7937b) |  | | 5.2 (5.76) | |  | | 159.11 | |  | | **9898a** |  | 5.2 (5.76) |  | 3 | 4.09 |
|  |  | 2006 |  |  |  |  | 77111 |  |  | OUTDOOR (VES) |  | T |  | 153.71-164.35 (149.71-169.35) |  | **9406b, 12722j, 10140e, 9898a, 2343e,** (13749d) |  | | 5.1 (5.45) | |  | | 160.35 | |  | | **2343e** |  | 5.1 (5.45) |  | 2.9 | 4.18 |
|  |  | 2005 |  |  |  |  |  | 2388 |  | INDOOR (AAFC) |  | A |  | 130.17-135.52 (122.81-135.52) |  | (5873t1, 14320e, 10140e, 12722j), **2343j, 7248f** |  | | 4.3 (4.69) | |  | | 134.73 | |  | | **13749k** |  | 4.3 (4.69) |  | 2.94 | 4.25 |
|  |  | 2006 |  |  |  |  |  | 2388 |  | INDOOR (AAFC) |  | T |  | 121.81-135.52 (118.38-135.52) |  | (10394t2), **5873t1, 14320e, 10140e, 12722j, 2343j, 13749k, 7248f** |  | | 4.1 (4.3) | |  | | 131.26 | |  | | **2343j** |  | 4 (4.27) |  | 2.87 | 4.2 |
|  |  | 2005 |  |  |  |  |  | 2388 |  | OUTDOOR (VES) |  | A |  | 124.05-135.52 (122.81-135.52) |  | (5873t1), **14320e, 10140e, 12722j, 2343j, 13749k, 7248f** |  | | 5.2 (5.59) | |  | | 131.26 | |  | | **2343j** |  | 4.9 (5.49) |  | 2.99 | 4.29 |
|  |  | 2006 |  |  |  |  |  | 2388 |  | OUTDOOR (VES) |  | T |  | 124.88-134.26 (122.81-135.52) |  | (5873t1,14320e), **10140e, 12722j, 2343j,** (13749k, 7248f) |  | | 4.6 (5.24) | |  | | 131.26 | |  | | **2343j** |  | 4.7 (5.21) |  | 2.82 | 4.29 |
|  |  |  |  |  |  |  |  |  |  |  |  |  |  |  |  |  |  | |  | |  | |  | |  | |  |  |  |  |  |  |
| **VIII** |  | 2006 |  |  |  |  | 77111 |  |  | Indoor (AAFC) |  | A |  | 118.48-149.78 (115.48-152.03) |  | **4142a, 15570a, 3191k, 1995a,** (10614t2, 16320b) |  | | 4.5 (4.15) | |  | | 130.39 | |  | | 15570a |  | 4.2 (4.05) |  | 3.07 | 4.18 |
|  |  | 2006 |  |  |  |  | 77111 |  |  | OUTDOOR (VES) |  | A |  | 124.74-152.03 (120.48-154.03) |  | (4142a), **15570a, 3191k, 1995a, 10614t2, 16320b** |  | | 5.2 (4.83) | |  | | 149.78 | |  | | **10614t2** |  | 4.2 (4.44) |  | 2.94 | 4.25 |
|  |  | 2006 |  |  |  |  |  | 2388 |  | Indoor (AAFC) |  | A |  | 104.87-129.46 (95.14-134.13) |  | (10616j, 8655e, 14232f, 2761e-2), **10695-1, 5515e, 3191k,** (8946a, 8885p2) |  | | 4.5 (3.56) | |  | | 114.64 | |  | | 10695-1 |  | 4.5 (3.56) |  | 2.93 | 4.25 |
|  |  | 2006 |  |  |  |  |  | 2388 |  | OUTDOOR (VES) |  | A |  | 109.87-130.46 (104.87-137.72) |  | **10695-1, 5515e, 3191k,** (8946a, 8885p2, 7016e, 3511e, 10231b, 5568j) |  | | 4.2 (4.32) | |  | | 123.46 | |  | | **3191k** |  | 3.9 (4.29) |  | 309 | 4.18 |
|  |  |  |  |  |  |  |  |  |  |  |  |  |  |  |  |  |  | |  | |  | |  | |  | |  |  |  |  |  |  |
| **X** |  | 2005 |  | 80112 |  |  |  |  |  | INDOOR (AAFC) |  | A |  | 30.51-60.54 (19.54-65.06) |  | (6491v1, 90003m, Contig-3389-814), **90019e, 10355, 5957t1, 8865, Contig-2443-400, 9121, 6349,** (Contig-4019-364) |  | | 10.5 (3.33) | |  | | 34.73 | |  | | 90019e |  | 9.3 (3.26) |  | 2.79 | 4.41 |
|  |  | 2005 |  |  | 80109 |  |  |  |  | INDOOR (AAFC) |  | A |  | 44.61-54.38 (20.44-66.92) |  | (8865), **Contig-28-365, 2603e, 9121, 6349,** (Contig-4019-364, Contig-556-520, 3814e) |  | | 9.3 (3.02) | |  | | 47.06 | |  | | Contig-28-365 |  | 8.5 (2.71 ns) |  | 2.75 | 4.56 |
|  |  | 2007 |  |  |  |  | 77111 |  |  | INDOOR (AAFC) |  | A |  | 1.20-29.10 (0.0-49.45) |  | (4794a, 6785c, sb31b, 12821a, 4341a), **6053b, 10364c, 7226a, 10408a, 10355v1,** (4543b, 8865a, 6593b, 9121f) |  | | 2.5 (2.73) | |  | | 10.39 | |  | | 10364c |  | 2.5 (2.73) |  | 2.4 | 4.2 |
|  |  | 2007 |  |  |  |  |  | 2388 |  | INDOOR (AAFC) |  | A |  | 0.0-8.93 (0.0-26.78) |  | **4914j, 4341e-2, 12821a, 4794f, 6053e, 5440e, 6903p1, 10698f, 8806a,** (90003m, 10355v3, 90019f) |  | | 4.7 (4.52) | |  | | 3.41 | |  | | **6053e** |  | 4.4 (4.45) |  | 2.78 | 4.29 |
|  |  | 2005 |  |  |  |  | 77111 |  |  | OUTDOOR (VES) |  | A |  | 84.24-130.97 (84.24-130.97) |  | **7914a, 7736b, 5437a, 1686c, 2871a, 8228m, 4148e, 5731j** |  | | 6.6 (2.42 ns) | |  | | 107.06 | |  | | 5437a |  | 4.4 (2.35 ns) |  | 2.52 | 4.09 |
|  |  | 2006 |  |  |  |  | 77111 |  |  | OUTDOOR (VES) |  | T |  | 84.24-130.97 (79.76-130.97) |  | **7914a, 7736b, 5437a, 1686c, 2871a, 8228m, 4148e, 5731j** |  | | 10.1 (3.11) | |  | | 84.26 | |  | | 7914a |  | 11 (2.99) |  | 2.52 | 4.18 |
|  |  | 2006 |  |  |  |  |  | 2388 |  | Indoor (AAFC) |  | T |  | 84.03-111.36 (47.48-111.36) |  | (9753e, 14356g), **2646e, 4334e, 11831f,** (9527h, 13366f, 7914h, 7235e)**, 7736e,** (10998e), **4348f, 10799e, 8228f, 3163e,** (13950e) |  | | 3.5 (3.07) | |  | | 106.16 | |  | | 10799e |  | 2.6 (2.81) |  | 2.72 | 4.2 |
|  |  | 2005 |  |  |  |  |  | 2388 |  | OUTDOOR (VES) |  | A |  | 59.17-82.39 (51.48-111.36) |  | (9753e, 14356g), **2646e, 4334e, 11831f, 9527h, 13366f, 7914h, 7235e, 7736e,** (10998e, 4348f, 10799e, 8228f, 3163e, 13950e) |  | | 2.8 (3.13) | |  | | 67.72 | |  | | 13366f |  | 2.8 (3.13) |  | 2.77 | 4.29 |
|  |  | 2006 |  |  |  |  |  | 2388 |  | Outdoor (VES) |  | T |  | 53.48- 83.03-111.36 (47.48-111.36) |  | **9753e, 14356g, 2646e, 4334e, 11831f, 9527h, 13366f, 7914h, 7235e, 7736e,** (10998e), **4348f, 10799e, 8228f, 3163e, 13950e** |  | | 2.7 (2.86 ns) | |  | | 58.17 | |  | | 14356g |  | 2.6 (2.6 ns) |  | 3.03 | 4.29 |

^a^ Two different colors of green distinguish the individual QTLs associated at two QTL-regions identified onto the same linkage group.

^b^ Years of measurement: 2004 and 2005 for the mapping population *P* and, 2006 and 2007 for the mapping population *D*.

^c^ Environmental conditions tested: in a large growth chamber (i.e. indoor conditions) at Agriculture and Agri-Food Canada (AAFC) under a declining photoperiod and day/night-time temperatures of 24/15°C; at Valcartier Experimental Station (VES) under natural outdoor conditions.

^d^ Measures of annual height growth (A) or total height growth (T).

^e^ Confidence interval of each QTL calculated by one-LOD and two-LOD below the QTL-LOD peak (i.e. at -1LOD and -2LOD).

^f^ Proportion of phenotypic variance explained.

^g^ ns = non significant.
